# Host Genetics of Response to Porcine Reproductive and Respiratory Syndrome in Sows: Reproductive Performance

**DOI:** 10.3389/fgene.2021.707870

**Published:** 2021-08-04

**Authors:** Felipe M. W. Hickmann, José Braccini Neto, Luke M. Kramer, Yijian Huang, Kent A. Gray, Jack C. M. Dekkers, Leticia P. Sanglard, Nick V. L. Serão

**Affiliations:** ^1^Department of Animal Science, Iowa State University, Ames, IA, United States; ^2^Department of Animal Science, Universidade Federal do Rio Grande do Sul, Porto Alegre, Brazil; ^3^Smithfield Premium Genetics, Rose Hill, NC, United States

**Keywords:** disease outbreak, genomics, GWAS, PRRS, QTL, reproduction, SNP, swine

## Abstract

Porcine Reproductive and Respiratory Syndrome (PRRS) is historically the most economically important swine disease worldwide that severely affects the reproductive performance of sows. However, little is still known about the genetic basis of reproductive performance in purebred herds during a PRRS outbreak through the comparison of maternal and terminal breeds. Thus, the objective of this work was to explore the host genetics of response to PRRS in purebred sows from two breeds. Reproductive data included 2546 Duroc and 2522 Landrace litters from 894 and 813 purebred sows, respectively, which had high-density genotype data available (29,799 single nucleotide polymorphisms; SNPs). The data were split into pre-PRRS, PRRS, and post-PRRS phases based on standardized farrow-year-week estimates. Heritability estimates for reproductive traits were low to moderate (≤0.20) for Duroc and Landrace across PRRS phases. On the other hand, genetic correlations of reproductive traits between PRRS phases were overall moderate to high for both breeds. Several associations between MARC0034894, a candidate SNP for response to PRRS, with reproductive performance were identified (*P*-value < 0.05). Genomic analyses detected few QTL for reproductive performance across all phases, most explaining a small percentage of the additive genetic variance (≤8.2%, averaging 2.1%), indicating that these traits are highly polygenic. None of the identified QTL within a breed and trait overlapped between PRRS phases. Overall, our results indicate that Duroc sows are phenotypically more resilient to PRRS than Landrace sows, with a similar return to PRRS-free performance between breeds for most reproductive traits. Genomic prediction results indicate that genomic selection for improved reproductive performance under a PRRS outbreak is possible, especially in Landrace sows, by training markers using data from PRRS-challenged sows. On the other hand, the high genetic correlations with reproductive traits between PRRS phases suggest that selection for improved reproductive performance in a clean environment could improve performance during PRRS, but with limited efficiency due to their low heritability estimates. Thus, we hypothesize that an indicator trait that could be indirectly selected to increase the response to selection for these traits would be desirable and would also improve the reproductive performance of sows during a PRRS outbreak.

## Introduction

Porcine Reproductive and Respiratory Syndrome (PRRS) is one of the most important swine diseases worldwide that affects the reproductive performance of sows and growth in young pigs. Some clinical signs of PRRS in sows include abnormal estrus cycle, late-term abortion, earlier farrow, and an increased number of stillbirths and mummified fetuses (Rossow et al., 1999; Lunney et al., 2011). The limited success in effectively controlling the PRRS virus (PRRSV) via traditional methods, such as vaccination and biosecurity procedures, has been reported to be due to the high mutation rate of PRRSV and the diversity of the strains circulating in the field (Brar et al., 2014; Montaner-Tarbes et al., 2019). Thus, exploring other methods, such as genetic and genomic selection, has been described as an additional and complementary tool to reduce the adverse effects caused by this pandemic (Dekkers et al., 2017).

Host genetics of response to PRRS in sows has been a subject of several studies over the last few years. These studies have indicated that reproductive performance traits in PRRSV-infected sows have low heritability (Lewis et al., 2009b; Rashidi et al., 2014; Serão et al., 2014; Putz et al., 2019; Scanlan et al., 2019). It has been shown that reproductive performance between healthy and PRRSV-infected animals is highly genetically correlated (Putz et al., 2019; Scanlan et al., 2019). On the other hand, Putz et al. (2019) also showed that the genetic correlation between reproductive performance prior to and after PRRSV infection in maternal breeds is low. This result indicates that reproductive performance in animals previously exposed to PRRSV may have a different genetic control than in naïve animals; however, this relationship has not been evaluated in other datasets, nor terminal breeds.

Studies on genomics of response to PRRS have provided information on major QTL and accuracies of genomic prediction. However, most of these studies focused on growing pigs (Boddicker et al., 2012; Dunkelberger et al., 2017; Waide et al., 2018). To the best of our knowledge, Serão et al. (2014) and Orrett (2017) are the only studies that provided GWAS results for reproductive traits in PRRSV-infected sows. Using Landrace sows under a PRRSV wild-type infection, Serão et al. (2014) reported a major QTL on *Sus scrofa* chromosome (SSC) 1, explaining 11% of the additive genetic variance for the number of stillborn piglets (NSB). However, these authors did not perform genomic prediction analyses for reproductive traits. Additional datasets must be evaluated to validate these previous results as well as to evaluate terminal lines, which have not yet been investigated. Genomic studies investigating genomic regions and genetic markers associated with reproductive performance across different PRRS phases are still a gap in the literature. Thus, the objectives of this work were to estimate genetic parameters of reproductive traits in sows before, during, and after a PRRS outbreak, perform genomic analyses for reproductive performance in PRRSV-infected purebred sows, and evaluate differences in PRRS resilience for reproductive performance between a terminal and a maternal breed.

## Materials and Methods

The data used for this study were collected as part of routine data recording in a commercial breeding program from a farm that operates in line with regulations on animal protection.

### Source of Data

Data were obtained from two commercial purebred populations (Duroc and Landrace) raised in the same farm separately, which experienced a PRRS outbreak during the Spring of 2018. Farrowing data included 2546 and 2522 litters from 894 Duroc and 813 Landrace sows, respectively, from June 2015 through July 2019. The Duroc and Landrace sows originated from 95 sires and 573 dams, and 114 sires and 502 dams, respectively. Traits used for this study were number of piglets born alive (NBA, pigs/litter), number of stillborn piglets (NSB, pigs/litter), number of mummified piglets (NBM, pigs/litter), number of piglets born dead (NBD, pigs/litter; the sum of NSB and NBM), total number of piglets born (TNB, pigs/litter; the sum of NBA and NBD), and number of piglets weaned (NW, pigs/litter). The net number of cross-fostered piglets (fostered in minus fostered out; XF) was also available. A total of 710 (28%) Duroc and 691 (27%) Landrace litters had cross-fostering. Prior to analyses, NSB, NBM, and NBD data were transformed as ln(phenotype+1) because of right skewness observed in the data (Serão et al., 2014). Table 1 shows the summary statistics of these traits by breed.

**TABLE 1:** Summary statistics of reproductive traits^1^ by breed.

	Duroc				Landrace			
		
Trait^2^	N^3^	Mean (SD)	Min	Max	N^3^	Mean (SD)	Min	Max
TNB	2511	8.61 (2.96)	3	19	2505	13.36 (4.00)	3	24
NBA	2546	7.41 (3.00)	0	17	2522	11.47 (4.08)	0	22
NBD	2511	1.15 (2.96)	0	15	2505	1.89 (2.78)	0	23
NSB	2546	0.62 (1.10)	0	15	2522	0.89 (1.43)	0	22
NBM	2511	0.52 (1.32)	0	15	2505	0.99 (2.21)	0	22
NW	2504	6.36 (3.07)	0	23	2476	9.04 (3.82)	0	26

*^1^Expressed as number of piglets/litter (standard errors in parenthesis);*

*^2^TNB, total number of piglets born; NBA, number of piglets born alive; NBD, number of piglets born dead; NSB, number of stillborn piglets; NBM, number of mummified piglets; NW, number of piglets weaned;*

*^3^Number of litters that have the trait recorded out of 2546 and 2522 litters from 894 Duroc and 813 Landrace sows, respectively.*

All animals had follicular hair or ear tissue samples taken and shipped to Neogen GeneSeek (Lincoln, NE, United States) for genotyping. Genotype data were available on all Duroc and Landrace sows for 33,776 and 39,610 SNPs, respectively. Genotypes were obtained using the GGP Porcine HD panel (Neogen GeneSeek) and processed according to the breeding company’s pipeline, which included removing non-segregating SNPs, SNPs with a minor allele frequency of less than 0.05, and minimum SNP call rate and animal call rate of 0.9. In addition, missing genotypes were imputed using Fimpute 2.2 (Sargolzaei et al., 2014). For subsequent analyses, only the 29,799 SNPs common to the genotype data from both breeds that passed quality control were used. The *Sscrofa* 11.1 assembly was used for the SNP location. The genotype data were used to construct a genomic relationship matrix for each breed separately based on VanRaden (2008), method 1. The wild-type PRRSV strain was sequenced and identified as PRRSV 1-7-4, a highly pathogenic strain.

### Identification of the PRRS Outbreak

The dataset was split into pre-PRRS, PRRS, and post-PRRS phases, following Putz et al. (2019), based on farrow-year-week (FYW) estimates (Lewis et al., 2009b). The FYW estimates were obtained for each breed from the following linear mixed model for reproductive traits, with the exception of NW:

(1) $$Y_{i\,j\,k}=\mu +P\,A\,R_{i}+f\,y\,w_{j}+s\,o\,w_{k}+e_{i\,j\,k}$$

where *Y*_*ijk*_ is the observed phenotype; μ is the general mean; *PAR*_*i*_ is the fixed effect of the *i*^*th*^ parity; *fyw*_*j*_ is the random effect of the *j*^*th*^ farrow-year-week, assuming $f\,y\,w_{j}∼N\,\left(0,\text{I}\,\sigma_{f\,y\,w}^{2}\right)$, where ***I*** is the identity matrix; *sow*_*k*_ is the random effect of sow, assuming $s\,o\,w_{k}∼N\,\left(0,\text{I}\,\sigma_{s\,o\,w}^{2}\right)$; and *e*_*ijk*_ is the random residual term associated with *Y*_*ijk*_, assuming $e_{i\,j\,k}∼N\,\left(0,\text{I}\,\sigma_{e}^{2}\right)$. For NW, the model above was modified to include the fixed effect covariate of XF. Analyses were performed with the package *lme4* (Bates et al., 2015) in R (R Core Team, 2017).

The FYW estimates were then standardized by their respective standard deviations (SDs) to make all traits comparable. Outbreaks of PRRS were identified for each trait separately to assess the disease’s impact on each reproductive trait, following Scanlan et al. (2019). For this, standardized FYW estimates that deviated 1.28 SD from the mean, representing a one-side probability threshold of 10%, were deemed extreme. The occurrence of two consecutive weeks of extreme values indicated the beginning of the PRRS phase. The end of the PRRS phase was defined by the return of standardized FYW estimates within 1.28 SD from the mean (i.e., from zero), followed by the occurrence of two consecutive weeks without extreme values. The pre-PRRS, PRRS, and post-PRRS phases were then defined accordingly for each reproductive trait. Not all animals experienced all three PRRS phases.

### Breed Effect on PRRS Resilience and Return to PRRS-Free Performance

The reproductive data from both breeds across all phases were used to evaluate how each breed was impacted by the PRRS outbreak. Since the average reproductive performance between Duroc and Landrace is quite different, the data were analyzed as a rate (i.e., proportion; described below) to allow for a fair comparison between the breeds. For each trait, two analyses were performed to identify the statistical method that best fit the data. Hence, the data from each trait was analyzed using Poisson and negative binomial mixed model methodologies, according to the following statistical model:

(2) $$\text{log}\,{\left(Y\right)}_{i\,j\,k\,l\,m}=\mu +B\,r\,e\,e\,d_{i}+P\,h\,a\,s\,e_{j}+{\left(B\,r\,e\,e\,d*P\,h\,a\,s\,e\right)}_{i\,j}+P\,A\,R_{k}+f\,y\,w_{l}+s\,o\,w_{m}+\text{log}\,{\left(T\right)}_{i\,j\,k\,l\,m}$$

where μ, *PAR*_*k*_, *fyw*_*l*_, and *sow*_*m*_ are as previously defined; log(*Y*)_*ijklm*_ is the log of the observed phenotype of the trait analyzed; *Breed*_*i*_ is the fixed effect of the *i*^th^ Breed (Duroc or Landrace); *Phase*_*j*_ is the fixed effect of the *j*^th^ Phase (pre-PRRS, PRRS, or post-PRRS); (Breed*Phase)_*ij*_ is the interaction between Breed and Phase; and log (*T*)_*ijklm*_ is the log of the trait used as the offset, described below.

The offset allowed the data to be analyzed as proportion, promoting fair comparison between breeds across phases. Depending on the trait analyzed, different offsets were used. TNB was used as the offset for all traits, with the exception of NW, as NW is also affected by XF. By using TNB as the offset, results represented the performance of the trait analyzed as a proportion of the litter size (i.e., TNB). The traits analyzed as proportion using TNB as an offset are referred to as NBA_TNB_, NBD_TNB_, NSB_TNB_, and NBM_TNB_. Two strategies were used in the analysis of NW. In the first, NW was analyzed using the sum of TNB and XF as the offset (NW_TNB,XF_). In this analysis, the proportion NW_TNB,XF_ represented the sow’s ability to wean all possible piglets that she could have farrowed (i.e., TNB) and had fostered in/out (i.e., XF). In the second strategy, NW was analyzed using the sum of NBA and XF as the offset (NW_NBA,XF_). In this analysis, the proportion NW_NBA,XF_ represented the sow’s ability to wean all possible piglets nursed by her [i.e., the opportunity piglets (i.e., NBA) and those that fostered in/out (i.e., XF)].

In order to evaluate the effect of breed on PRRS resilience and on return to PRRS-free performance, two pre-defined contrasts were used when the effect of the interaction between Breed and Phase was significant (*P*-value ≤ 0.05). With the levels of the interaction denoted as (1) Duroc-pre-PRRS, (2) Landrace-pre-PRRS, (3) Duroc-PRRS, (4) Landrace-PRRS, (5) Duroc-post-PRRS, and (6) Landrace-post-PRRS, the following two contrasts were evaluated:

i. *PRRS resilience*, with coefficients of 1, −1, −1, 1, 0, and 0 for the six respective interaction levels. In this contrast, we evaluated the difference in the decline in relative reproductive performance from the pre-PRRS to the PRRS phase between the two breeds;
ii. *Return to PRRS-free performance*, with coefficients of 1, −1, 0, 0, −1, and 1 for the six respective interaction levels. In this contrast, we evaluated the difference in the rate of return to PRRS-free performance between the two breeds. In other words, we compared whether the relative reproductive performances between the pre-PRRS and post-PRRS phases were the same for both breeds.

Significance was declared at *P*-value ≤ 0.05, and a trend was declared at 0.05 < *P*-value < 0.10. For completeness, Tukey-Kramer separation was performed if the interaction between breed and phase was significant. Prior to the final analyses, for each trait, the dispersion parameter estimated using the negative binomial model was tested again at a value of 1 (representing a Poisson model) using a likelihood ratio test. Analyses indicated that the dispersion parameter was significant for NBD_TNB_, NSB_TNB_, NBM_TNB_, and NW_NBA,XF_, and hence, a negative binomial model was used for these traits. In contrast, NBA_TNB_ and NW_TNB,XF_ were analyzed using a Poisson model. Analyses were performed in SAS 9.4 (SAS Institute Inc., Cary, NC, United States).

### Genetic Parameters

Heritabilities and genetic correlations were estimated separately for each breed and phase. Genetic correlations were estimated between phases within traits. Genetic correlations between traits within phases were not estimated because bivariate analyses within the PRRS phase had convergence issues due to the low sample size. For the pre-PRRS and post-PRRS phases, the following model was used to estimate heritabilities:

(3) $$Y_{i\,j\,k\,l}=\mu +P\,A\,R_{i}+f\,y\,w_{j}+a_{k}+p\,e_{k}+e_{i\,j\,k}$$

where *Y*_*ijk*_, μ, *PAR*_*i*_, *fyw*_*j*_, and *e*_*ijk*_ are as previously defined; *a_k_* is the animal genetic random effect, assuming $a_{k}∼N\,\left(0,\text{GRM}\,\sigma_{a}^{2}\right)$, where **GRM** is the genomic relationship matrix; and *pe*_*k*_ is the random permanent environment effect, assuming $p\,e_{k}∼N\,\left(0,\text{I}\,\sigma_{p\,e}^{2}\right)$. For NW, this model was modified to include the number of net cross-fostered piglets as a covariate. For the PRRS phase, *pe* and *fyw* were removed from the model as only one observation per animal was available for this phase. The bivariate model used to estimate the genetic correlations between phases within traits included the same effects as the univariate model to estimate heritabilities according to the respective PRRS-phase being considered in the analysis. All analyses were performed in ASReml v4 (Gilmour et al., 2015).

### Effect of SNPs Previously Associated With Response to PRRS

The effects of the MARC0034894/rs80841011 (1:28,912,680) (MARC) and WUR10000125/rs80800372 (4:127,441,677) (WUR) SNPs, which were previously associated with NSB in PRRSV-infected Landrace sows (Serão et al., 2014) and with viremia and growth rate in PRRSV-infected nursery pigs (Boddicker et al., 2012, 2014b), respectively, were investigated by simultaneously fitting them as fixed effects in the model used for estimation of genetic parameters described above. Animals with the *BB* genotype for the WUR SNP were combined with those with the *AB* genotype due to the dominance mode-of-action described for this SNP (Boddicker et al., 2014b). Analyses were performed separately for each PRRS phase and breed. All analyses were performed in ASReml v4 (Gilmour et al., 2015).

### Genome-Wide Association Studies

Genome-wide association studies (GWAS) were performed separately for each PRRS phase and breed, using the BayesB method with π = 0.99 (Habier et al., 2011). Pre- and post-PRRS data were pre-adjusted for fixed effects due to repeated records on the same individuals. In other words, the adjusted phenotype (*y*^∗^) was obtained for each animal as the sum of the estimated random animal effect, permanent environmental effect, and the average residuals from the model used for the estimation of genetic parameters. For the GWAS, residuals for a given trait were weighted based on the number of records on each animal and trait parameter estimates, with weights derived as in Garrick et al. (2009):

(4) $$w_{n}=\frac{1-h^{2}}{c\,h^{2}+\frac{1+\left(n-1\right)\,t}{n}-h^{2}}$$

where *w*_*n*_ represents the weighing factor for *n* observations; *h*^2^ is the estimated heritability of the trait; *c* is the proportion of the genetic variance not accounted for by markers, which was assumed to be 0.75 for all traits; and *t* is the estimated repeatability of the trait. GWAS models for pre- and post-PRRS included only the intercept as fixed effect, with residuals being weighted according to the values obtained with the formula above, and the random allele substitution effects of SNPs. For the PRRS phase, the same models previously described for the PRRS phase were used but replacing the animal genetic effect by the random allele substitution effects of SNPs. For all analyses, additive genetic and residual variances obtained from the genetic parameter analyses were used as priors. A total of 50,000 Markov Chain Monte Carlo (MCMC) iterations were used, of which the first 10,000 iterations were used as burn-in. All analyses were performed using GenSel version 4.4 (Fernando and Garrick, 2009). Consecutive 1-Mb genomic regions that explained at least 0.5% of the total additive genetic variance accounted for by markers (TGVM) were combined. In the end, genomic regions that explained more than 1% of TGVM were deemed significant and further investigated to identify candidate genes. For the presentation of GWAS results, the start of the QTL region on a given SSC c was assumed to be c:Mbi,000,000, and the end of the QTL region as c:Mbf,999,999 where Mbi and Mbf represent the Mb where the identified QTL window started and ended, respectively. Thus, for example, if a QTL was identified in a given 1-Mb region r, the position of the QTL was expressed as rMb, such that Mbi = Mbf = r and the QTL encompassed c:r,000,000–r,999,999. In contrast, when closely located 1-Mb QTL regions were combined into a single window, the position of the QTL was expressed as r-r’Mb, such that Mbi = r < Mbf = r’ and the QTL encompassed c:r,000,000–r’,999,999.

### Genomic Prediction

Genomic prediction accuracies (GPA) were obtained using the same model as described for GWAS but using BayesB (π = 0.99), BayesC (π = 0.99), and BayesC0 (BayesC with π = 0), separately for each breed and trait. The overall objective of these analyses was to predict the performance of PRRSV-infected sows since information on GPA in PRRSV-infected sows in the literature is limited.

Five genomic prediction scenarios (GPS) were investigated according to different strategies used for the training datasets (i.e., the dataset used to estimate SNP effects). The training datasets differed according to the combination between the source of the dataset used for training (pre-PRRS phase and/or PRRS phase) and whether or not animals in the validation dataset were included in the training dataset. These five GPS are summarized below and in Table 2. In all GPS, data from the PRRS phase was used as the validation dataset. When multiple sources of data were used for training, estimation of SNP effects was performed within each source of the data. In other words, estimation of marker effects was obtained separately using data from the pre-PRRS and PRRS phases.

**TABLE 2:** Summary of genomic prediction scenarios (GPS) evaluated.

	Training datasets^1^			Validation dataset (PRRS)^2^		Calculation of GEBVs^3,4^	
			
	Pre-PRRS		PRRS (Folds)				
	
Scenario	All	Folds		All	Folds	All	Folds
GPS_PRRS_	−	−	✓	−	✓		✓
GPS_pre–PRRS_	✓	−	−	✓	−	✓	
GPS_pre–PRRS–4FCV_	−	✓	−	−	✓		✓
GPS_pre–PRRS,PRRS_	✓	−	✓	−	✓	½	½
GPS_pre–PRRS–4FCV,PRRS_	−	✓	✓	−	✓	½	½

*^1^Source of data used in the training dataset. In GPSs including both phases (pre-PRRS and PRRS), marker effects were estimated separately for each source of data;*

*^2^In all analyses, data from the PRRS phase was used for validation;*

*^3^Genomic estimated breeding values (GEBVs);*

*^4^In the last two GPSs (GPS_*pre–PRRS,PRRS*_ and GPS_*pre–PRRS–4FCV,PRRS*_), the GEBV of each individual was calculated as the average GEBV obtained from the marker effects estimates using each training set (i.e., pre-PRRS and PRRS phases).*

1. GPS_PRRS_: The training dataset included data from the PRRS phase only. In order to avoid using the same animal in the training and validation datasets, analyses were performed using a 4-fold cross-validation (4FCV). Thus, genomic estimated breeding values (GEBVs) in the validation set were calculated per fold. Details about the generation of the 4 folds are included below.
2. GPS_pre–PRRS_: The training dataset included data from the pre-PRRS phase only. This approach was used since the two phases do not co-exist at the same time. Hence, in practice, GEBVs in the validation set (i.e., during PRRS) could be obtained using pre-PRRS data, in which the same animals are used in the training and validation datasets.
3. GPS_pre–PRRS–4FCV_: The training dataset included only data from the pre-PRRS phase. In this GPS, we modified GPS_pre–PRRS_ to represent cases where animals have data in one of the phases only (i.e., pre-PRRS or PRRS phase). Hence, GEBVs in the validation set were calculated per fold.
4. GPS_pre–PRRS,PRRS_: The training datasets included data from both the pre-PRRS and PRRS phases. Since the two phases do not co-exist at the same time, all the pre-PRRS data were used. However, in order to avoid using the same animals in the PRRS phase for training and validation, the PRRS dataset was subjected to a 4FCV. Hence, GEBVs in the validation set were obtained as the average GEBV obtained from training SNPs using the pre-PRRS and PRRS phases.
5. GPS_pre–PRRS–4FCV,PRRS_: The training dataset included data from both the pre-PRRS and PRRS phases. This strategy is a modification of the previous scenario (GPS_pre–PRRS,PRRS_), in which a 4FCV was used for datasets. Hence, GEBVs in the validation set were obtained as the average GEBV obtained from training SNPs using the pre-PRRS and PRRS phases.

The folds used in the 4FCV analyses were created by randomly assigning sows from the same sire family to one of the four folds. This strategy was used to increase the relatedness of individuals between folds, which is expected in traditional breeding schemes. Then, three folds were used for training and the remaining fold for validation. This process was repeated until all four folds were used for validation. The number of records per fold, trait, and breed is presented in Supplementary Table 1. These folds were created using the PRRS data only, as this was the target dataset for prediction purposes. However, some animals in the pre-PRRS phase did not have data in the PRRS. Therefore, these animals were always used in the training datasets but never in the validation datasets. The number of records per fold differed between traits because the timing of the PRRS phase differed between traits.

The genomic prediction accuracy (GPA) for scenarios using Folds (i.e., all GPSs except GPS_pre–PRRS_) were calculated as a weighted average as:

(5) $$G\,P\,A=\frac{\sum_{i=1}^{4}n_{i}\,r_{i}\,\left(G\,E\,B\,V,y^{*}\right)}{\sqrt{h^{2}}}$$

where *r*_*i*_(*GEBV*,*y*^∗^) is the correlation between GEBVs and the phenotypes adjusted for fixed effects (*y*^∗^) in the *i*^*th*^ validation dataset, which was weighted by the proportion of records in the validation dataset of each fold (*n*_*i*_); and *h*^2^ is the estimate of heritability of the trait being analyzed during the PRRS phase. The GPA of the GPS using all the data from the pre-PRRS phase (i.e., GPS_pre–PRRS_) was obtained as where $r\,\left(G\,E\,B\,V,y^{*}\right)/{\sqrt{h}}^{2}$, where all terms are as previously defined. Estimation of marker effects were obtained in GenSel v.4.4 (Fernando and Garrick, 2009).

## Results

### Identification of the PRRS Outbreak

**FIGURE 1:**
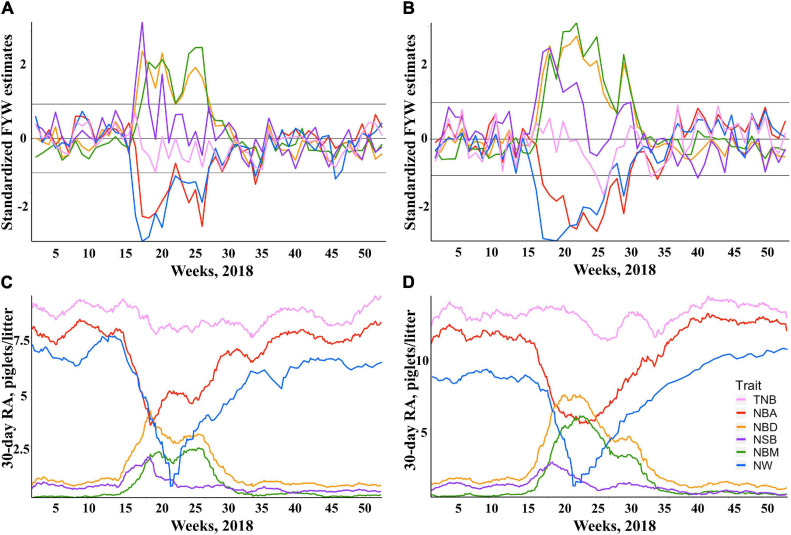
Impact of Porcine Reproductive and Respiratory Syndrome (PRRS) on herd average reproductive performance. Standardized estimates of farrow-year-week (FYW) during 2018 for each reproductive trait for Duroc **(A)** and Landrace **(B)** sows. Thirty-day rolling averages (RA) of reproductive traits for Duroc **(C)** and Landrace **(D)** sows.

The standardized FYW estimates and 30-d RAs for all traits are shown in Figure 1 for both breeds. The extreme increase (over the 90^th^ percentile) in standardized FYW estimates for NBD, NBM, and NSB, and an extreme decrease (under the 10^th^ percentile) in standardized FYW estimates for NBA and NW were evident for both breeds in the same period as shown in Figures 1A,B for Duroc and Landrace, respectively. From these results, the beginning of the PRRS phase was set to be the 15^th^ week of 2018 for all traits. All data prior to this date were defined as the pre-PRRS phase. The end of the PRRS phase was characterized by the return of standardized FYW estimates to be close to 0, which differed between traits. For mortality traits (NBD, NBM, and NSB), the end of the PRRS phase was set to be the 30^th^ week of 2018 for both breeds, while for NBA, NW, and TNB, the end of the PRRS phase was set to be the 34^th^ week of 2018 for both breeds. Visually, the same reduction pattern and return to normal production were observed for both breeds (Figures 1C,D for Duroc and Landrace, respectively). The summary statistics by phase and breed are shown in Table 3.

**TABLE 3:** Summary statistics of reproductive traits^1^ by PRRS^2^ phase and breed.

	Duroc				Landrace			
		
Trait^3^	N^4^	Mean (SD)	Min	Max	N^4^	Mean (SD)	Min	Max
**Pre-PRRS phase**								
TNB	1004 (478)	8.94 (2.88)	3	19	1096 (461)	13.36 (3.79)	3	24
NBA	1004 (478)	7.90 (2.67)	0	18	1096 (461)	11.81 (3.33)	0	22
NBD	978 (468)	1.03 (1.37)	0	13	1073 (450)	1.55 (1.87)	0	18
NSB	978 (468)	0.66 (1.02)	0	9	1073 (450)	1.01 (1.44)	0	13
NBM	978 (468)	0.37 (0.80)	0	10	1073 (450)	0.54 (0.99)	0	13
NW	1004 (478)	6.98 (2.51)	0	24	1096 (461)	9.37 (2.96)	0	26
**PRRS phase**								
TNB	494 (494)	7.89 (3.13)	3	19	429 (429)	12.59 (3.98)	3	24
NBA	501 (501)	5.50 (3.30)	0	15	432 (432)	7.53 (4.73)	0	19
NBD	494 (494)	1.40 (1.19)	0	15	429 (429)	3.24 (1.47)	0	23
NSB	501 (501)	0.59 (0.75)	0	12	432 (432)	0.93 (0.90)	0	12
NBM	494 (494)	0.75 (1.11)	0	15	429 (429)	1.99 (1.61)	0	22
NW	501 (501)	3.92 (3.19)	0	12	432 (432)	4.84 (4.15)	0	14
**Post-PRRS phase**								
TNB	1028 (542)	8.63 (3.03)	3	17	980 (513)	13.75 (3.82)	3	24
NBA	1028 (542)	7.82 (2.79)	0	17	980 (513)	12.55 (3.46)	0	22
NBD	1079 (558)	0.78 (1.19)	0	15	1025 (527)	1.26 (1.96)	0	23
NSB	1079 (558)	0.50 (0.92)	0	15	1025 (527)	0.75 (1.47)	0	22
NBM	1079 (558)	0.28 (0.68)	0	9	1025 (527)	0.51 (1.19)	0	22
NW	1028 (542)	6.69 (3.21)	0	23	980 (513)	10.05 (3.59)	0	26

*^1^Expressed as number of piglets/litter (standard errors in parenthesis);*

*^2^Porcine Reproductive and Respiratory Syndrome (PRRS);*

*^3^TNB, total number of piglets born; NBA, number of piglets born alive; NBD, number of piglets born dead; NSB, number of stillborn piglets; NBM, number of mummified piglets; NW, number of piglets weaned;*

*^4^Number of litters (number of sows).*

### Breed Effect on PRRS Resilience and Return to PRRS-Free Performance

Results for these analyses are presented in Table 4. With the exception of NSB_TNB_ (*P*-value = 0.300), there was a significant (*P*-value ≤ 0.026) interaction between PRRS phase and breed for all traits. For traits with this significant interaction, all traits but NSB_TNB_ (*P*-value = 0.161) and NW_NBA, XF_ (*P*-value = 0.127) had a significant (*P*-value ≤ 0.039) *PRRS resilience* contrast. Results showed that, proportionally, the drop in reproductive performance from the pre-PRRS to the PRRS phase was greater in Landrace than in Duroc sows. Prior to the PRRS outbreak, Duroc and Landrace sows had proportionally similar (*P*-value > 0.05) NBA_TNB_ and NBM_TNB_, with 0.866 ± 0.013 and 0.882 ± 0.011 NBA_TNB_, respectively, and 0.039 ± 0.003 and 0.034 ± 0.003 NBM_TNB_, respectively. However, during the PRRS phase, Duroc sows had, proportionally, better reproductive performance (*P*-value < 0.05) than Landrace sows, with 0.676 ± 0.017 and 0.590 ± 0.014 NBA_TNB_, respectively and 0.150 ± 0.017 and 0.232 ± 0.025 NBM_TNB_, respectively. Although Landrace had proportionally lower (*P*-value < 0.05) NBD_TNB_ (0.106 ± 0.005) than Duroc sows (0.122 ± 0.007) prior to the PRRS outbreak, the relationship inverted during the PRRS phase, where Duroc sows had lower (*P*-value < 0.05) NBD_TNB_ (0.299 ± 0.023) than Landrace (0.396 ± 0.030). Interestingly, Duroc had greater (*P*-value < 0.05) NW_TNB, XF_ than Landrace sows in both pre-PRRS and PRRS phases. However, this superiority was more evident in the PRRS phase. Prior to the PRRS outbreak, the NW_TNB, XF_ of Duroc and Landrace sows were 0.750 ± 0.019 and 0.700 ± 0.016, respectively, whereas in the PRRS phase, these were 0.371 ± 0.018 and 0.322 ± 0.015, respectively.

**TABLE 4:** Effect^1^ of PRRS^2^ phase and breed on reproductive traits.^3^

*Phase*	*Breed*	NBA_TNB_	NBD_TNB_	NSB_TNB_	NBM_TNB_	NW_TNB, XF_	NW_NBA, XF_
Pre-PRRS	Duroc	0.866^b^(0.013)	0.122^c^(0.007)	0.083^c^(0.005)	0.039^b^(0.003)	0.750^a^(0.019)	0.787^a^(0.021)
	Landrace	0.882^ab^(0.011)	0.106^b^(0.005)	0.070^b^(0.004)	0.034^ab^(0.003)	0.700^bc^(0.016)	0.716^bc^(0.018)
PRRS	Duroc	0.676^c^(0.017)	0.299^d^(0.023)	0.125^d^(0.011)	0.150^c^(0.017)	0.371^d^(0.018)	0.342^d^(0.017)
	Landrace	0.590^d^(0.014)	0.396^e^(0.030)	0.124^d^(0.011)	0.232^d^(0.025)	0.322^e^(0.015)	0.294^e^(0.015)
Post-PRRS	Duroc	0.895^ab^(0.014)	0.093^ab^(0.005)	0.063^b^(0.004)	0.030^a^(0.003)	0.722^ab^(0.024)	0.742^ab^(0.026)
	Landrace	0.905^a^(0.012)	0.087^a^(0.005)	0.053^a^(0.003)	0.033^ab^(0.003)	0.689^c^(0.023)	0.705^c^(0.023)
***ANOVA P-values***							
Breed		0.001	0.483	0.011	0.010	<0.001	<0.001
Phase		<0.001	<0.001	<0.001	<0.001	<0.001	<0.001
Breed*Phase		<0.001	<0.001	0.300	<0.001	0.026	0.018
***Contrast P-values^4^***							
Resilience		<0.001	<0.001	0.161	<0.001	0.039	0.127
Return to PRRS-free performance		0.712	0.329	0.969	0.103	0.307	0.073

*^1^Results represented as proportions based on total number of piglets born (TNB), with or without the number of cross-fostered pigs (XF), or number of piglets born alive (NBA) with XF. Standard errors in parenthesis;*

*^2^Porcine Reproductive and Respiratory Syndrome (PRRS);*

*^3^NBA_*TNB*_, NBA with TNB used as the offset; NBD_*TNB*_, number of piglets born dead (NBD) with TNB used as the offset; NSB_*TNB*_, number of stillborn piglets (NSB) with TNB used as the offset; NBM_*TNB*_, number of mummified piglets (NBM) with TNB used as the offset; NW, number of piglets weaned; NW_*TNB*,*XF*_, NW with the sum of TNB and the net number of cross-fostered pigs (XF) used as the offset; NW_*NBA,XF*_, NW with the sum of NBA and XF used as the offset;*

*^4^Resilience, contrast representing the differences between breeds for the difference between pre-PRRS and PRRS; Return to PRRS-free performance, contrast representing the differences between breeds for the difference between pre-PRRS and post-PRRS;*

*^*a*–*e*^Means lacking the same superscript within a column indicate differences at *P*-value < 0.05.*

The *return to PRRS-free performance* contrast had a trend effect only for NW_NBA, XF_ (*P*-value = 0.073). Although there were no differences in NW_NBA, XF_ within breed between pre-PRRS and post-PRRS (*P*-value > 0.05), the *return to PRRS-free performance* contrast indicated that NW_NBA, XF_ tended to have a greater reduction in Landrace sows from pre-PRRS to post-PRRS (0.716 ± 0.018 to 0.705 ± 0.023, respectively) than in Duroc sows (0.787 ± 0.021 to 0.742 ± 0.026, respectively). In both phases, Duroc had greater (*P*-value < 0.05) NW_NBA, XF_ than Landrace sows. Overall, these results indicate that Duroc sows have greater PRRS resilience than Landrace sows.

### Genetic Parameters

Heritability (h^2^) estimates for reproductive traits were low to moderate across datasets, as shown in Table 5. Overall, there was no consistency of estimates across PRRS phases for a given trait. Nonetheless, as expected, h^2^ estimates were overall low for all traits, breeds, and phases. From the pre-PRRS to the PRRS phase, there was a numerical increase in estimates of additive genetic variances for litter mortality traits (i.e., NDB, NSB, and NBM) in both breeds. In contrast, residual variance estimates numerically increased during the PRRS phase for all traits and breeds. Most estimates of the additive genetic variance were numerically greater in the post-PRRS phase than in the pre-PRRS phase, while residual variance estimates were numerically lower in the post-PRRS than in the PRRS phase.

**TABLE 5:** Estimates of genetic parameters^1,2^ for reproductive traits by PRRS^3^ phase and breed.

	Pre-PRRS phase			PRRS phase			Post-PRRS phase		
			
Trait^4^	h^2^ (SE)	$\sigma_{a}^{2}$	$\sigma_{e}^{2}$	h^2^ (SE)	$\sigma_{a}^{2}$	$\sigma_{e}^{2}$	h^2^ (SE)	$\sigma_{a}^{2}$	$\sigma_{e}^{2}$
**Duroc**									
TNB	0.01 (0.01)	1.08	6.48	0.11 (0.07)	1.03	8.38	0.15 (0.04)	1.36	7.49
NBA	0.01 (0.02)	1.36	5.60	0.12 (0.07)	1.33	9.56	0.13 (0.04)	1.01	6.66
NBD	<0.01 (0.01)	<0.01	0.29	0.09 (0.06)	0.05	0.54	0.11 (0.03)	0.03	0.24
NSB	<0.01 (0.01)	0.01	0.21	0.02 (0.05)	0.01	0.30	0.07 (0.03)	0.01	0.18
NBM	0.01 (0.02)	<0.01	0.13	0.02 (0.05)	0.01	0.53	0.06 (0.03)	0.01	0.11
NW	0.06 (0.03)	0.36	3.37	0.12 (0.06)	1.20	9.08	0.14 (0.04)	1.07	5.97
**Landrace**									
TNB	0.05 (0.02)	1.82	12.63	<0.01 (0.05)	0.07	15.32	0.20 (0.04)	2.76	10.74
NBA	0.07 (0.02)	1.57	9.16	0.06 (0.06)	1.45	20.83	0.16 (0.04)	1.94	9.66
NBD	<0.01 (0.01)	0.02	0.35	0.13 (0.08)	0.10	0.71	0.10 (0.03)	0.03	0.32
NSB	0.01 (0.01)	0.03	0.25	0.16 (0.08)	0.06	0.35	0.12 (0.03)	0.03	0.22
NBM	0.02 (0.02)	<0.01	0.18	0.08 (0.07)	0.07	0.84	0.01 (0.02)	<0.01	0.19
NW	0.12 (0.03)	0.51	5.10	0.08 (0.07)	1.40	15.87	0.13 (0.04)	1.30	8.29

*^1^Heritability, h^2^; additive genetic variance, $\sigma_{a}^{2}$; residual variance, $\sigma_{e}^{2}$;*

*^2^Expressed as piglets^2^ for TNB, NBA, and NW, and ln(piglets+1)^2^ for the other traits;*

*^3^Porcine Reproductive and Respiratory Syndrome (PRRS);*

*^4^TNB, total number of piglets born; NBA, number of piglets born alive; NBD, number of piglets born dead; NSB, number of stillborn piglets; NBM, number of mummified piglets; NW, number of piglets weaned.*

Estimates of genetic correlations (r_g_) of each reproductive trait between the three phases are shown in Table 6. These estimates varied considerably between phases within the same trait, with large standard errors. Nonetheless, estimates were all positive. Between the pre-PRRS and PRRS phases, r_g_ estimates ranged from 0.06 ± 0.42 (TNB) to 0.94 ± 0.56 (NW) for Duroc, and from 0.47 ± 0.83 (NBA) to 0.84 ± 0.35 (NBD) for Landrace. Estimates of r_g_ between the pre-PRRS and post-PRRS phases ranged from 0.33 ± 0.46 (NSB) to 0.90 ± 0.38 (NW) for Duroc, and from 0.69 ± 0.63 (TNB) to 0.90 ± 0.47 (NBD) for Landrace. However, r_g_ estimates for NBA, NSB, and NW in Landrace, and for NBD in Duroc did not converge. Estimates of r_g_ between the PRRS and post-PRRS phases ranged from 0.10 ± 0.49 (NBA) to 0.94 ± 0.44 (NW) for Duroc, and from 0.10 ± 0.31 (NSB) to 0.96 ± 0.30 (TNB) for Landrace.

**TABLE 6:** Estimates of genetic correlations (SE) of reproductive traits between PRRS^1^ phases by breed.

Trait^2^	Pre-PRRS/PRRS	Pre-PRRS/Post-PRRS	PRRS/Post-PRRS
**Duroc**			
TNB	0.06 (0.42)	0.85 (0.36)	0.81 (0.26)
NBA	0.73 (0.30)	0.87 (0.36)	0.10 (0.49)
NBD	0.38 (0.36)	NC^3^	0.71 (0.19)
NSB	0.73 (0.97)	0.33 (0.46)	NC^3^
NW	0.94 (0.56)	0.90 (0.38)	0.94 (0.44)
**Landrace**			
TNB	0.70 (0.68)	0.69 (0.63)	0.96 (0.30)
NBA	0.47 (0.83)	NC^3^	0.68 (0.42)
NBD	0.84 (0.35)	0.90 (0.47)	0.31 (0.33)
NSB	0.83 (0.22)	NC^3^	0.10 (0.31)
NW	0.73 (0.53)	NC^3^	0.93 (0.47)

*^1^Porcine Reproductive and Respiratory Syndrome (PRRS).*

*^2^TNB, total number of piglets born; NBA, number of piglets born alive; NBD, number of piglets born dead; NSB, number of stillborn piglets; NW, number of piglets weaned.*

*^3^NC, not converged. Number born mummified (NBM) did not converge for both breeds.*

### Effect of SNPs Previously Associated With Response to PRRS

In this study, only a few associations of the MARC and WUR SNPs with reproductive traits were identified (Table 7). The only association (*P*-value = 0.037) of the WUR SNP with reproductive performance in Landrace sows was found for pre-PRRS NW, where *AA* animals had greater (9.61 ± 0.20) performance than *AB* animals (9.23 ± 0.24). For Duroc sows, there was a trending association (*P*-value = 0.095) of the WUR SNP with pre-PRRS NW, with *AA* animals also showing greater performance (7.32 ± 0.22) than *AB* animals (7.05 ± 0.19).

**TABLE 7:** Least squares means^1^ (SE) for reproductive traits across PRRS^2^ phases for genotypes at the WUR10000125 and MARC0034894 SNP^3^ in Duroc and Landrace sows.

	WUR10000125			MARC0034894			
		
Trait^4^	*AA*	*AB*	*P*-value	*AA*	*AB*	*BB*	*P*-value
**Pre-PRRS phase**							
*Duroc*							
TNB	9.62 (0.29)	9.38 (0.24)	0.308	9.70 (0.35)	9.50 (0.26)	9.29 (0.25)	0.386
NBA	8.46 (0.28)	8.22 (0.23)	0.290	8.61 (0.34)	8.30 (0.25)	8.10 (0.24)	0.273
NBD^5^	0.77 (0.05)	0.79 (0.04)	0.794	0.72 (0.06)	0.81 (0.05)	0.80 (0.04)	0.633
NSB^5^	0.52 (0.05)	0.53 (0.04)	0.847	0.50 (0.06)	0.54 (0.04)	0.53 (0.04)	0.832
NBM^5^	0.23 (0.04)	0.24 (0.03)	0.788	0.20 (0.04)	0.24 (0.03)	0.25 (0.03)	0.574
NW	7.32^A^(0.22)	7.05^B^(0.19)	0.095	7.33 (0.26)	7.17 (0.20)	7.06 (0.19)	0.465
*Landrace*							
TNB	13.58 (0.30)	13.31 (0.36)	0.365	13.12 (0.58)	13.75 (0.30)	13.48 (0.27)	0.428
NBA	12.15 (0.26)	11.84 (0.31)	0.236	11.71 (0.51)	12.27 (0.27)	12.01 (0.24)	0.393
NBD^5^	1.01 (0.04)	1.07 (0.05)	0.508	1.03 (0.09)	1.03 (0.04)	1.06 (0.04)	0.949
NSB^5^	0.72 (0.04)	0.72 (0.05)	0.899	0.73 (0.08)	0.70 (0.04)	0.73 (0.03)	0.934
NBM^5^	0.27 (0.03)	0.32 (0.04)	0.189	0.27 (0.06)	0.30 (0.03)	0.30 (0.03)	0.918
NW	9.61^a^(0.20)	9.23^b^(0.24)	0.037	8.96^b^(0.37)	9.80^a^(0.21)	9.50^ab^(0.18)	0.033
**PRRS phase**							
*Duroc*							
TNB	8.73 (0.37)	8.72 (0.29)	0.969	8.99 (0.47)	8.76 (0.32)	8.42 (0.32)	0.372
NBA	5.49 (0.37)	5.44 (0.30)	0.861	5.35 (0.48)	5.56 (0.32)	5.49 (0.32)	0.898
NBD^5^	1.91 (0.08)	2.02 (0.07)	0.613	2.04 (0.11)	2.01 (0.07)	1.84 (0.07)	0.654
NSB^5^	0.65 (0.06)	0.76 (0.05)	0.266	0.65 (0.08)	0.70 (0.05)	0.77 (0.05)	0.624
NBM^5^	1.21 (0.08)	1.18 (0.06)	0.826	1.43 (0.10)	1.16 (0.07)	1.02 (0.07)	0.184
NW	3.68 (0.40)	3.68 (0.32)	0.990	3.79 (0.48)	3.56 (0.35)	3.69 (0.36)	0.837
*Landrace*							
TNB	12.51 (0.34)	12.24 (0.41)	0.484	11.74^b^(0.72)	12.27^b^(0.36)	13.12^a^(0.29)	0.033
NBA	7.69 (0.40)	7.38 (0.48)	0.507	8.26^A^(0.83)	6.75^B^(0.41)	7.60^A^(0.34)	0.077
NBD^5^	3.16 (0.07)	3.17 (0.09)	0.980	2.29^B^(0.15)	3.61^AB^(0.07)	3.76^A^(0.06)	0.055
NSB^5^	1.02 (0.05)	1.00 (0.07)	0.870	0.87 (0.11)	1.05 (0.06)	1.11 (0.05)	0.571
NBM^5^	1.82 (0.07)	1.84 (0.09)	0.945	1.16^b^(0.16)	2.14^ab^(0.08)	2.33^a^(0.06)	0.027
NW	5.11 (0.33)	4.89 (0.41)	0.562	5.60 (0.67)	4.44 (0.35)	4.96 (0.30)	0.158
**Post-PRRS phase**							
*Duroc*							
TNB	8.95 (0.25)	8.93 (0.18)	0.946	9.58^a^(0.35)	8.70^ab^(0.21)	8.54^b^(0.20)	0.023
NBA	8.00 (0.23)	7.99 (0.17)	0.969	8.70^a^(0.32)	7.74^ab^(0.20)	7.53^b^(0.19)	0.003
NBD^5^	0.63 (0.04)	0.65 (0.03)	0.784	0.62 (0.06)	0.66 (0.03)	0.64 (0.03)	0.900
NSB^5^	0.45 (0.03)	0.44 (0.03)	0.770	0.41 (0.05)	0.46 (0.03)	0.46 (0.03)	0.733
NBM^5^	0.16 (0.03)	0.19 (0.02)	0.363	0.21 (0.04)	0.17 (0.02)	0.16 (0.02)	0.688
NW	6.80 (0.27)	6.72 (0.22)	0.740	7.29^A^(0.35)	6.51^AB^(0.23)	6.47^B^(0.23)	0.055
*Landrace*							
TNB	13.73 (0.24)	13.63 (0.29)	0.745	13.86 (0.51)	13.38 (0.25)	13.79 (0.20)	0.312
NBA	12.46 (0.23)	12.34 (0.27)	0.656	12.63 (0.47)	12.10 (0.24)	12.45 (0.20)	0.302
NBD^5^	0.87 (0.04)	0.84 (0.04)	0.771	0.82 (0.08)	0.86 (0.04)	0.89 (0.03)	0.833
NSB^5^	0.53 (0.03)	0.53 (0.04)	0.977	0.53 (0.07)	0.52 (0.03)	0.54 (0.03)	0.945
NBM^5^	0.29 (0.02)	0.29 (0.03)	0.953	0.24 (0.05)	0.31 (0.03)	0.32 (0.02)	0.599
NW	9.95 (0.23)	9.85 (0.26)	0.710	10.24 (0.44)	9.84 (0.23)	9.63 (0.20)	0.371

*^1^Expressed as number of piglets;*

*^2^Porcine Reproductive and Respiratory Syndrome (PRRS);*

*^3^SNPs are located on *Sus scrofa* chromosome 7 according to the *Sscrofa* 11.1 as follow: (GCA_000003025.6) assembly: WUR10000125/rs80800372 (4:127,441,677) and MARC0034894/rs80841011 (1:28,912,680). Frequencies of the *A* and *B* alleles for the WUR10000125 SNP were 0.51 and 0.49, respectively, for Duroc, and 0.81 and 0.19, respectively, for Landrace, while for the MARC0034894 SNP were 0.31 and 0.69, respectively, for Duroc, and 0.25 and 0.75, respectively, for Landrace for the PRRS-phase;*

*^4^TNB, total number of piglets born; NBA, number of piglets born alive; NBD, number of piglets born dead; NSB, number of stillborn piglets; NBM, number of mummified piglets; NW, number of piglets weaned;*

*^5^Results were back-transformed from ln(phenotype+1);*

*^*a,b*^Means lacking the same superscript indicate differences at *P*-value < 0.05 between genotypes with an SNP;*

*^*A,B*^Means lacking the same superscript indicate differences at *P*-value < 0.10 between genotypes with an SNP.*

Many more associations were found for the MARC SNP, in particular for Landrace sows. In the pre-PRRS phase, this SNP was associated (*P*-value = 0.033) with NW, where *AB* sows (9.8 ± 0.21) weaned more (*P*-value < 0.05) piglets than *AA* sows (8.96 ± 0.37), with both not differing (*P*-value > 0.05) from *BB* sows (9.50 ± 0.18). During the PRRS phase, MARC SNP was associated with the reproductive performance of most traits in Landrace. For TNB (*P*-value = 0.033), *BB* (13.12 ± 0.29) animals had greater (*P*-value < 0.05) performance than *AA* (11.74 ± 0.72) and *AB* (12.27 ± 0.36). Interestingly, for NBA (*P*-value = 0.077), there were no differences (*P*-value > 0.10) between *AA* (8.26 ± 0.83) and *BB* (7.60 ± 0.34) animals, although both genotypes had greater (*P*-value < 0.10) NBA than *AB* sows (6.75 ± 0.41). For NBD (*P*-value = 0.055) and NBM (*P*-value = 0.027), the same pattern was observed, with better performance increasing with the number of the *A* allele. Sows with genotype *AA* had better NBD (*P*-value < 0.10) and NBM (*P*-value < 0.05), with 2.29 ± 0.15 and 1.16 ± 0.16, respectively, than *BB* sows (3.76 ± 0.06 and 2.33 ± 0.06, respectively). For both traits, *AB* sows did not differ in NBD (3.61 ± 0.07; *P*-value > 0.10) and NBM (2.14 ± 0.08; *P*-value < 0.05) from the other genotypes. No associations (*P*-value ≥ 0.302) were found between the MARC SNP and reproductive performance post-PRRS in Landrace sows.

In contrast, the MARC SNP was only associated with post-PRRS performance in Duroc sows. Associations were found for TNB (*P*-value = 0.023), NBA (*P*-value = 0.003), and NW (*P*-value = 0.055). In all associations, better performance was observed as the number of *A* alleles increased. Sows with the *AA* genotype had greater TNB (9.58 ± 0.35), NBA (8.70 ± 0.32), and NW (7.29 ± 0.35) than *BB* sows, who had 8.54 ± 0.20, 7.53 ± 0.19, and 6.47 ± 0.23, respectively. These did not differ from sows with *AB* genotype.

### Genomic Regions Associated With Reproductive Traits

Genomic regions that explained more than 1% of TGVM in reproductive performance across PRRS phases are displayed in Table 8. In general, these QTL explained a low %TGVM of the traits. For Duroc pre-PRRS, there were nine QTL identified, with two for TNB, NBA, NBD, and NW, and one for NBM. Of these, the largest QTL was identified for NBA on SSC 7 (31–33 Mb), close to the MHC region, explaining 2.8% TGVM. For Duroc PRRS, there were four QTL identified, one for each trait (TNB, NBA, NSB, and NW). The largest QTL was found for TNB on SSC 5 (36–41 Mb), explaining 7.2% TGVM. For Duroc post-PRRS, there were seven QTL identified, with three for TNB, two for NBA, and one for NBM and NW. The largest QTL was identified for NBA (8.2% TGVM) on SSC 11 (22 Mb), which was also identified for TNB (2.0% TGVM) and NW (1.4% TGVM).

**TABLE 8:** Genomic regions associated^1^ with reproductive performance across PRRS^2^ phases for Duroc and Landrace sows.

Trait^3^	%TGVM	SSC	Mb	#SNPs
**Pre-PRRS phase**				
*Duroc*				
TNB	1.3	6	41–42	30
	1.2	14	103–104	29
NBA	2.8	7	31–33	46
	1.2	16	70–73	61
NBD	1.8	4	114–116	63
	1.0	5	83–86	36
NBM	1.6	9	120–123	61
NW	1.3	15	119	35
	2.2	15	125–129	152
*Landrace*				
TNB	1.4	5	4–8	176
	1.5	9	8–10	89
	1.1	16	2–5	55
NBA	1.3	5	7–10	108
	4.3	9	8–10	89
	1.4	16	2–5	55
NSB	7.4	6	41–43	39
NBM	1.2	6	0–2	54
	1.1	15	119–122	97
**PRRS phase**				
*Duroc*				
TNB	7.2	5	36–41	39
NBD	2.0	13	189–190	43
NSB	1.2	5	5–9	131
NW	2.9	13	189–190	21
*Landrace*				
NBA	1.2	10	7–9	50
	1.5	13	156–160	40
NBD	1.2	3	13–15	112
	1.0	9	11–13	90
NBM	1.1	9	11–13	90
**Post-PRRS phase**				
*Duroc*				
TNB	1.9	4	86–87	36
	1.2	9	128–130	92
	2.0	11	22	21
NBA	1.9	9	128–131	115
	8.2	11	22	21
NBM	1.8	2	46–47	21
NW	1.4	11	22	21
*Landrace*				
TNB	1.7	8	111–113	37
	1.3	12	55–56	35
NBA	1.3	2	11–12	54
	2.9	8	111–113	37
NW	3.1	3	1–2	49

*^1^Genomic regions explaining more than 1% of the total additive genetic variance accounted for by markers (%TGVM). Traits not included on the table did not have any genomic region explaining more than 1% of TGVM;*

*^2^Porcine Reproductive and Respiratory Syndrome (PRRS);*

*^3^TNB, total number of piglets born; NBA, number of piglets born alive; NBD, number of piglets born dead; NBM, number of piglets born mummified; NW, number of piglets weaned; SSC, *Sus scrofa* chromosome; Mb, location of the SNP window within the SSC, in megabases; #SNPs, number of SNPs in the region.*

For Landrace pre-PRRS, there were eight QTL identified, three for TNB and NBA, two for NBM, and one for NSB. The largest identified QTL was for NSB on SSC 6 (41–43 Mb), explaining 7.4% TGVM. TNB and NBA shared two QTL: one on SSC 9 (8–10 Mb), explaining 1.5 and 4.3% TGVM for TNB and NBA, respectively, and one on SSC 16 (2–5 MB), explaining 1.1 and 1.4% TGVM for TNB and NBA, respectively. In addition, a QTL on SSC 5 was identified for these two traits without a complete overlap between the QTL regions of these traits, on 4–8 Mb (1.4% TGVM) for TNB and on 7–10 Mb (1.3% TGVM) for NBA. For Landrace PRRS, five QTL were identified, with two for NBA and NBD, and one for NBM. The largest QTL was for NBA on SSC 13 (156–160 Mb), explaining 1.5% TGVM. For Landrace post-PRRS, there were five QTL identified, with the largest QTL (SSC 3, 1–2 Mb) explaining 3.1% TGVM for NW. TNB and NBA had the same QTL on SSC 8 (111–113 Mb), explaining 1.7 and 2.9% TGVM, respectively. Although many QTL were identified, they were not consistent across traits and phases within a breed. Several candidate genes were identified in these regions and will be discussed below.

### Genomic Prediction Accuracies

**FIGURE 2:**
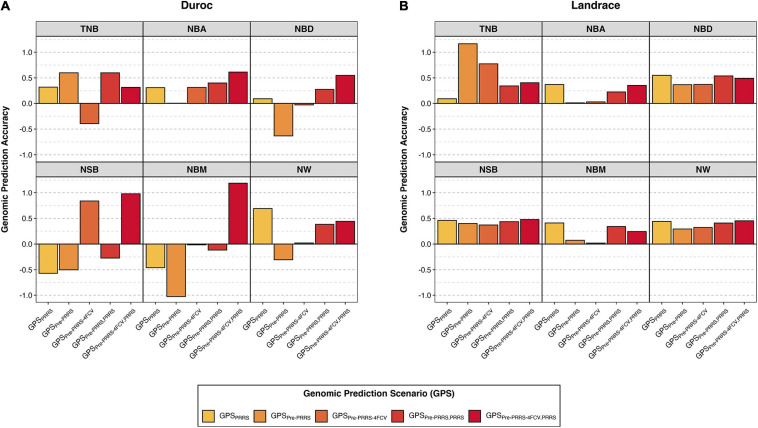
Genomic prediction accuracies of reproductive traits during a Porcine Reproductive and Respiratory Syndrome (PRRS) outbreak. Results are presented for Duroc **(A)** and Landrace **(B)** across genomic prediction scenarios (GPS) for total number born (TNB), number born alive (NBA), number born dead (NBD), number of stillborn (NSB), number born mummified (NBM), and number of piglets weaned (NW) using BayesB. The *y*-axis represents the genomic prediction accuracy and the *x*-axis the GPS. Details for the different GPS are available in Table 2. Results for all methods and standard deviations across folds are available in Supplementary Table 2.

Overall, genomic prediction accuracies (GPAs) were similar across Bayesian methods, and thus, results presented in the main text are just for one method (BayesB). These are shown in Figure 2, whereas results from all methods are available in Supplementary Table 2. In general, there was no consistency in GPAs between traits, breeds, and GPSs. In general, GPAs were better in Landrace than in Duroc.

In Duroc, although there was considerable variation in GPAs across GPSs within a trait, in general, results obtained from scenarios combining data from the pre-PRRS and PRRS phases for training yielded better GPAs. Among the given GPSs, GPS_pre–PRRS–4FCV,PRRS_ is the only scenario that resulted in positive GPAs for all traits. In addition, this GPS yielded the highest GPAs (SD across the four folds) for NBA (0.61 ± 0.48), NBD (0.55 ± 0.73), NSB (0.98 ± 2.05), and NBM (1.19 ± 2.55). For TNB, the highest GPAs were obtained in scenarios GPS_pre–PRRS_ (0.60) and GPS_pre–PRRS,PRRS_ (0.60 ± 0.12), whereas for NW, the highest GPA was obtained in GPS_PRRS_ (0.69 ± 0.06). However, some negative GPAs were obtained in these analyses. Of these, large negative GPAs (<−0.3) were obtained using GPS_PRRS_ for NSB (−0.57 ± 0.04) and NBM (−0.46 ± 0.12), GPS_pre–PRRS_ for NBD (−0.63), NSB (−0.50), NBM (−1.03), and NW (−0.31), and GPS_pre–PRRS–4FCV_ for TNB (−0.39 ± 0.6).

In contrast, all GPAs were positive in Landrace. Results across GPSs within a trait were similar, with the exception of TNB. For this trait, the highest GPA was obtained using GPS_pre–PRRS_ (1.16), although a high GPA was also obtained using GPS_pre–PRRS–4FCV_ (0.77 ± 1.95). Interestingly, GPS_PRRS_ showed the lowest GPA for TNB (0.09 ± 0.09), whereas for the other traits, this GPS yielded the highest or second highest GPAs. GPS_PRRS_ had the highest GPAs for NBA (0.37 ± 0.03), NBD (0.55 ± 0.12), and NBM (0.41 ± 0.14). For NSB, the GPA for GPS_PRRS_ was 0.46 ± 0.08, whereas the highest GPA was obtained in GPS_pre–PRRS–4FCV,PRRS_, with 0.48 ± 0.29. Finally, for NW, the GPA for GPS_PRRS_ was 0.44 ± 0.14, whereas the highest GPA was also obtained in GPS_pre–PRRS–4FCV,PRRS_, with 0.45 ± 0.31. Overall, the GPAs in Landrace were better and more consistent across GPS and traits than in Duroc. In general, combining data from the pre-PRRS and PRRS phases did not substantially yield better GPAs in Landrace.

## Discussion

### Identification of the PRRS Outbreak

We used standardized FYW estimates to identify when the PRRS outbreak occurred to split the reproductive data into three different datasets. Although the beginning of the PRRS phase was set to be April 9^th^, 2018, animals were probably infected with PRRSV prior to that date, before the reproductive performance of sows was affected. Increases in abortions and piglet mortality traits, such as NSB and NBM, are usually reported as the first clinical signs of a PRRS outbreak (Rossow et al., 1999; Lunney et al., 2011). There was an increased incidence of mortality traits under PRRS for both breeds, which reinforced the severity of the disease. Survival traits, such as NBA and NW, had a decrease in means during the PRRS phase, which is in line with what other studies had previously found using this approach (Serão et al., 2014; Putz et al., 2019; Scanlan et al., 2019). With the exception of TNB, all traits showed improved mean performance after the outbreak, reaching similar performance to the production levels prior to the outbreak.

### Breed Effect on PRRS Resilience and Return to PRRS-Free Performance

Breed differences play an important role when it comes to PRRS-resilience. Many studies have reported that growing pigs from lines selected for improved reproductive performance (e.g., Landrace, Meishan, Large White) are more resilient to the effects of PRRS than pigs from lines selected for carcass traits and growth (e.g., Duroc, Pietran) because of the severe effects of a PRRSV infection on the lungs of animals selected for lean growth (Halbur et al., 1998; Petry et al., 2005; Vincent et al., 2006). On the other hand, Lewis et al. (2009a) reported that Meishan sows, commonly selected for improved reproductive performance and maternal ability, had greater susceptibility to PRRS than sows from terminal lines.

We evaluated how proportionally each breed changed its performance between PRRS phases to evaluate the impact of breed on PRRS resilience and on return to PRRS-free performance. For this, we performed analyses using an offset, so the count data for each trait would be adjusted to its total count (TNB for most traits). This approach was used to allow a fair comparison between breeds, as their performance is different since Landrace animals are selected to have improved reproductive performance, whereas Duroc is used as a terminal line. Results from these analyses indicated that Duroc has greater PRRS resilience than Landrace sows.

For most traits, the decrease in performance from pre-PRRS to PRRS was lower in Duroc than in Landrace sows. For instance, the decrease in NBA_TNB_ was 21.9 ± 2.2% in Duroc and 33.1 ± 1.7% in Landrace. As expected, this reduction in NBA_TNB_ due to the PRRS outbreak was accompanied by an increase in piglet mortality traits for both breeds. There was an increase in NBD_TNB_ of 144.7 ± 22.4% in Duroc and 275.1 ± 32.4% in Landrace sows. For both breeds, this increase in NBD_TNB_ was driven by an increase in NBM since there was a significant difference in NBM_TNB_ and not in NSB_TNB_ for the *PRRS resilience* contrast. NBM_TNB_ increased by 285.6 ± 52.6% in Duroc and 575.6 ± 86.4% in Landrace sows from pre-PRRS to PRRS.

An increase in NBD is one of the traditional signs of a PRRS outbreak in a commercial farm (Rossow et al., 1999; Lunney et al., 2011). Depending on the timing of PRRSV infection during pregnancy, sows are expected to show differences in NSB and NBM. As shown in Figures 1C,D for Duroc and Landrace, respectively, there was a numerically greater average of NSB than NBM within the first 6 weeks of the PRRS phase. This is expected, as it indicates that potentially viable piglets had recently died in the uterus due to the PRRSV infection. In contrast, NBM increased after 6 weeks, as they died during pregnancy at earlier development stages, resulting in their mummification. In addition, the distribution of farrowing events was very similar between Duroc and Landrace over the PRRS period. About 25% of the farrowing events from each breed occurred within the first 6 weeks of the PRRS phase. Hence, the lack of significant *PRRS resilience* contrast effect for NSB_TNB_ should be due to the clear effect of PRRSV infection during the first weeks, without significant differences between breeds. In contrast, due to the delayed effect on performance, our analyses were powerful enough to detect differences in PRRS resilience for NBM_TNB_.

Among all traits evaluated, NW was the only one in which two approaches were used. In NW_TNB,XF_, we evaluated the weaning performance of sows with respect to her maximum biological limit to produce piglets (i.e., TNB). Similar to the results presented for the other traits, from pre-PRRS to PRRS, Duroc sows had a lower reduction in NW_TNB,XF_ than Landrace sows, with reductions of 50.6 ± 2.5 and 54.1 ± 2.3%, respectively. However, the same was not observed for NW_NBA,XF_, in which the *PRRS resilience* contrast was not significant, although, numerically, there was a lower reduction observed in Duroc (56.6 ± 2.2%) than in Landrace (59.0 ± 2.2%). In NW_NBA,XF_ we evaluated the weaning performance of sows with respect to her realized potential to produce piglets (i.e., NBA). In other words, in NW_NBA,XF_ we considered only the opportunity piglets she could have weaned, as those that were born dead could not have been weaned by her. This lack of significant *PRRS resilience* contrast for NW_NBA,XF_ could be due to the significant breed effect in NBA_TNB_, indicating that, proportionally, the two breeds differ in NBA. Hence, by using NBA as part of the offset for NW, the difference in NBA_TNB_ should have removed the breed difference for NW_NBA,XF_. Thus, the different results obtained in NW_TNB,XF_ and NW_NBA,XF_ for the *PRRS resilience* contrast indicate a breed difference in perinatal (i.e., TNB) resilience, rather than resilience from farrowing to weaning.

Results suggest that Duroc sows have overall greater PRRS resilience for reproductive traits than Landrace sows. The applicability of these results for the industry, however, is limited since commercial sows are usually Landrace x Large White crosses. Nevertheless, if these traits are genetically correlated with terminal traits, such as feed efficiency, commercial hogs may benefit from this overall superiority observed in Duroc sows since these hogs are usually made up of 50% Duroc. These results further suggest that Duroc sows have lower drop in reproductive performance than Landrace sows from pre-PRRS to PRRS. Second, our analyses did not consider within-breed genetic effects due to the overall small sample size for genetic analyses using generalized models. Although we were able to identify differences in reproductive performance between the two breeds across PRRS phases, by not fitting a random animal effect in the model, these results were not adjusted for within-breed differences, nor the degrees of freedom of the test statistics evaluated were corrected by the complex pedigree relationships. Nonetheless, breed differences are due to genetic factors. Thus, the phenotypic superiority of Duroc sows compared to Landrace sows with regards to PRRS resilience should be due to the genetic make-up of these animals.

### Genetic Parameters

Ranges of h^2^ estimates for reproductive traits in this study were consistent with previous estimates found for healthy and PRRSV-infected sows (Lewis et al., 2009b; Serão et al., 2014; Putz et al., 2019; Scanlan et al., 2019). For most traits, h^2^ estimates for litter mortality traits were higher during the PRRS outbreak. Putz et al. (2019) suggested that the increased incidence of these traits could explain these higher h^2^ estimates during the PRRS phase. In most cases, this increase in h^2^ estimates was accompanied by an increase in the estimate of additive genetic variance. This increase was much clearer in Landrace sows than in Duroc ones. The increase in additive genetic variance for mortality traits from the pre-PRRS to the PRRS phase observed in this study for Landrace sows is in accordance with the literature (Serão et al., 2014; Putz et al., 2019), even in F1 (Landrace x Large White) sows (Scanlan et al., 2019). In Duroc, estimates of additive genetic variance for NBA and TNB were similar across phases. This aligns with the overall greater phenotypic resilience observed in this study for Duroc sows.

It is expected that the additive genetic variance of traits that have been selected in a clean and healthy environment will be higher in diseased animals compared to healthy animals (Berghof et al., 2019). Terminal lines such as Duroc are selected for higher feed efficiency, carcass, and growth traits, not for maternal traits, in contrast to Landrace (Bishop et al., 2010). In this study, the presence of this pattern, however, varied between breeds and traits. For Duroc, the estimates for TNB and NBA were very similar across phases, whereas, for NBD, NSB, NBM, and NW, these estimates substantially increased from the pre-PRRS to the PRRS phase, and then decreased during the post-PRRS phase. For Landrace, estimates of additive genetic variances increased from the pre-PRRS to the PRRS phase for NBD, NSB, NBM, and NW too. During the post-PRRS phase, these estimates generally decreased for these traits.

Most studies that included r_g_ estimates between PRRS phases partitioned data into only two phases (healthy and disease phases), combining data from prior to and after the outbreak as one phase only (Lewis et al., 2009b; Rashidi et al., 2014; Scanlan et al., 2019). On the other hand, Putz et al. (2019) reported r_g_ estimates between traits across different PRRS phases (pre-PRRS, PRRS, and post-PRRS). We also split data into three phases to better understand changes over time, to analyze how litter size traits eventually return to their production levels after the outbreak, and to identify differences between breeds in their ability to recover from the PRRS outbreak. Putz et al. (2019) and Scanlan et al. (2019) have shown that the reproductive performance of healthy and PRRSV-infected sows is highly genetically correlated. The r_g_ estimates between litter mortality traits in this study prior to and during a PRRSV infection were consistent with those previous findings. These results suggest that selecting animals in a clean environment for improved reproductive performance before an outbreak would also improve the reproductive performance of animals infected with PRRSV. However, h^2^ estimates for reproductive performance are still low, and the use of an indicator trait to indirectly increase response to selection for these traits would be desirable.

Putz et al. (2019) estimated low r_g_ between reproductive performance prior to and after a PRRSV infection in maternal breeds. They also indicated that the reproductive performance in healthy sows previously exposed to PRRSV might have a different genetic control than in naïve animals. In contrast, we found much higher r_g_ estimates between survival traits prior to and after a PRRSV infection than Putz et al. (2019) for both breeds, suggesting that reproductive traits in naïve animals and healthy animals after infection share a common genetic control. These conflicting results indicate that additional studies are needed to understand this relationship better. Nonetheless, in our study and in Putz et al. (2019), the standard errors associated with estimates of genetic correlation were large, suggesting that results might not be real. Some r_g_ estimates for litter mortality traits between PRRS phases had convergence issues in our study, partially explained by the low sample size and the large standard errors.

Overall, these results indicate that selection for improved reproductive performance during a PRRS outbreak is possible, but with limited efficiency because of the low heritability estimates of these traits, regardless of the PRRS phase. Therefore, the identification of an indicator trait, such as antibody response to PRRSV as proposed by Serão et al. (2014), would greatly benefit the swine industry to accelerate the rate of genetic improvement for these traits under a PRRS outbreak. Antibody response to PRRSV, measured as S/P ratio, was shown to be moderately heritable in Landrace and Duroc sows during a PRRS outbreak. In combination with the high genetic correlation between S/P ratio and NBA in Landrace (0.61) and the negative genetic correlations with mortality traits, Hickmann et al. (2021) validated the use of S/P ratio as an indicator trait for improved reproductive performance under a PRRS outbreak in Landrace populations. In addition, Sanglard et al. (2020) demonstrated that antibody response to PRRSV vaccination in gilts is highly genetically correlated with subsequent reproductive performance in the absence of a PRRS outbreak. Nonetheless, the high genetic correlations between PRRS phases suggest that selection for improved reproductive performance in a clean environment (i.e., in the absence of PRRS) could result in improved response during a PRRS outbreak, but with limited efficiency due to their low heritability estimates. In addition, the large standard errors associated with these estimates must be taken into consideration.

### Effect of SNPs Previously Associated With Response to PRRS

The WUR SNP on SSC 4 has been associated with PRRS tolerance in growing pigs, in which *AB* piglets had favorable performance compared to those with the *AA* genotype (Boddicker et al., 2012; Hess et al., 2018). Serão et al. (2014) identified associations (*P*-value ≤ 0.057) between WUR genotype and NBA and NW during the pre-PRRS phase in an outbreak study, with *AB* sows having better performance than *AA* sows. In our study, the only association (*P*-value = 0.037) between the genotype at WUR SNP and reproductive performance was found for pre-PRRS NW in Landrace sows. Contrary to Serão et al. (2014), *AA* animals had greater performance (9.61 ± 0.20) than *AB* (9.23 ± 0.24) animals. Serão et al. (2014) did not find associations (*P*-value > 0.10) within the PRRS phase, neither did we (*P*-value ≥ 0.266). In our study, there were no associations (*P*-value ≥ 0.363) between the WUR SNP and reproductive performance in both Duroc and Landrace sows during the post-PRRS phase. Although the effect of the WUR SNP has been well validated in multiple studies in PRRSV-exposed growing pigs (Abella et al., 2016; Dunkelberger et al., 2017; Hess et al., 2018), its association with reproductive traits is limited in the literature. It could be that its effect on these traits is very small or, in fact, not existing. Our results could suggest the latter, although a much larger sample size might be needed to better understand this relationship, and we cannot accept the null hypothesis of lack of associations. Finally, with the large number of comparisons performed in this study for this marker (2 breeds × 3 phases × 6 traits = 36 tests), which was not accounted for in the significance tests, the association with NW in Duroc during the pre-PRRS phase could be a false positive.

Serão et al. (2014) found an association (*P*-value < 0.001) between the MARC SNP on SSC 1 with NSB in reproductive sows during the PRRS phase, with *BB* sows showing favorable performance. In our study, there were no associations between this SNP and NSB (*P*-value = 0.571). However, there were associations with other reproductive traits (TNB, NBM, NBA, and NBD) in Landrace sows during the PRRS phase. As in Serão et al. (2014), we also found favorable associations for sows with the *BB* genotype for the MARC SNP. With the exception of NBA, in which *AA* (8.26 ± 0.83) and *BB* (7.60 ± 0.34) animals had greater performance than *AB* (6.75 ± 0.41) sows, greater performance in TNB, NBM, and NBD was obtained as the number of the *B* allele increased in Landrace sows. There were no associations with reproductive traits in Duroc sows during the PRRS phase. This lack of associations could be because Duroc sows are selected for different traits than Landrace sows, and thus, the linkage disequilibrium between this marker and the QTL might be weak. On the other hand, during the post-PRRS phase, there were significant associations (*P*-value ≤ 0.055) between the MARC SNP and reproductive traits (TNB, NBA, and NW) for Duroc sows but not for Landrace sows. Interestingly, these associations for Duroc were not found during the pre-PRRS phase, although pre-PRRS traits were highly genetically correlated with the corresponding post-PRRS traits. Furthermore, the QTL that harbors this SNP on SSC1 for NSB during the PRRS phase in Serão et al. (2014) was not identified in this study for any of the traits, further supporting that this region might not be important in the populations used in our study. Altogether, the MARC SNP seems to have a much greater potential to be used as a genetic marker for improved reproductive performance than the WUR SNP. Nonetheless, the significant associations observed for the MARC SNP in this independent dataset bring new possibilities for marker-assisted selection for improved reproductive performance under a PRRS outbreak in Landrace sows or following a PRRS outbreak in Duroc sows. Further research is needed to pinpoint the reasons for the opposite results in these two populations, while focusing on identifying the quantitative trait nucleotide responsible for this effect.

### Genome-Wide Association Studies

Reports on GWAS for reproductive traits in PRRSV-infected sows are scarce in the literature. Most studies have performed GWAS analyses to investigate genomic regions associated with host response to experimental PRRSV infection in growing pigs (Boddicker et al., 2012, 2014a,b; Waide et al., 2018). These studies have provided information about major QTL associated with viremia and weight gain in pigs. Lewis et al. (2009c) reported SNPs associated with reproductive traits during a PRRS outbreak in sows but did not report the specific genomic regions associated with these traits. Orrett (2017) also reported several QTL associated with reproductive performance in PRRSV-infected sows: on SSC 1 (220–226 Mb) for NBM, on SSC 5 (89–93 Mb), SSC 6 (78–80 Mb), and SSC 9 (127–137) for NSB, on SSC 10 (69–70 Mb) for NBD, and on SSC 3 (28–30 Mb), SSC 4 (137–140 Mb), SSC 7 (107–113 Mb), and SSC 8 (26–28 Mb) for NBA. None of these genomic regions were identified in our study. Serão et al. (2014) reported a QTL on SSC 1 (32–35 Mb) that explained 11% of TGVM for NSB and 1% TGVM for NBD during the PRRS phase in Landrace sows. In our study, there were no QTL associated with NSB in the PRRS phase in Landrace, but we did find a QTL for this trait in Duroc sows (Table 8).

We also found other QTL associated with reproductive traits during the PRRS outbreak in both breeds that were not previously reported. Two QTL appeared to be associated with more than one trait: the QTL on SSC 13 (189–190 Mb) that was associated with NBD and NW in Duroc sows, and the QTL on SSC 9 (11–13 Mb) that was associated with NBD and NBM in Landrace sows. The 6-Mb region on SSC 5 associated with TNB in Duroc sows has not previously been associated with reproductive traits in sows. This region had the largest %TGVM in this study, with 7.2%. Two candidate genes in this region play a role in reproduction; the GLIPR1-like protein 1 gene (*GLIPR1L1*) involved with fertilization, with a potential role in sperm-oocyte binding (Gibs et al., 2010), and the GLIPR1-like protein 2 gene (*GLIPR1L2*) that plays a role in a great variety of processes, including immune response and membrane development (Ren et al., 2006). Zhang et al. (2019) reported two QTL on SSC 5 (9 and 67 Mb) associated with litter size traits at birth in non-PRRS-infected Duroc sows. These two regions are in a different position than the 36–41 Mb region associated with TNB in our study; however, the QTL located at 9 Mb overlaps with the region associated with NSB in our study for Duroc. Four genes in this 1-Mb interval are related to reproductive development and energy metabolism that may play a role during a viral infection. The apolipoprotein B mRNA editing enzyme catalytic subunit 3B gene (*APOBEC3B*) acts as an inhibitor of retrovirus replication and retrotransposon mobility. This gene protects the cell or organism in the presence of a virus with species-specific interactions (Schröfelbauer et al., 2004). The Eukaryotic translation initiation factor 3 subunit L gene (*EIF3L*) plays a role in the process of viral translational termination-reinitiation and is required for several steps in the initiation of protein synthesis (Masutani et al., 2007; Lee et al., 2015). Both Platelet-derived growth factor subunit B (*PDGFB*) and SRY-box transcription factor 10 (*SOX10*) genes are also located within this 1-Mb region and regulate embryonic development, being involved in the cell response to growth factor stimulus as well (Sekido and Lovell-Badge, 2009). Another region including a reproductive-related gene is the 3-Mb region on SSC 10 associated with NBA in Landrace sows, which harbors a gene associated with spermatogenetic failures, the spermatogenesis associated 17 gene (*SPATA17*; Deng et al., 2006). We did not find any candidate genes that play a role in reproduction within the QTL on SSC 14 (125–126 Mb) for NW in Duroc sows, or within the QTL on SSC 13 (156–160 Mb) for NBA in Landrace sows.

A large number of QTL have been reported in the literature for reproductive traits in non-infected pigs (Onteru et al., 2011; Verardo et al., 2016; Suwannasing et al., 2018). These QTL considerably varied depending on the trait being considered. We identified several QTL associated with reproductive traits for the pre-PRRS phase that were not previously reported. Two QTL were associated with two traits: the QTL on SSC 9 (8–10 Mb) and the QTL on SSC 16 (2–5 Mb), both of them associated with TNB and NBA in Landrace sows. Other QTL associated with more than one trait had some overlapping regions, such as the QTL on SSC 5 (4–8 Mb) and the QTL on SSC 5 (7–10 Mb) associated with TNB and NBA, respectively, in Landrace sows. Interestingly, the same genomic regions controlling TNB were also associated with NBA in Landrace sows. The QTL found on SSC 15 (119 Mb) for NW in Duroc sows was also found in Landrace sows, however, for NBM. In this region, there is a candidate gene that plays a role in reproduction: the transition protein 1 gene (*TNP1*) involved with spermatogenesis in mammals (Khattri et al., 2011). Another region including a reproductive-related gene is the 3-Mb region on SSC 7 (31–33 Mb) close to the MHC region that was associated with NBA in Duroc sows during the pre-PRRS phase, which harbors a gene associated with sperm capacitation, the T-complex protein 11 gene (*TCP11*; Castaneda et al., 2020).

Several QTL with relatively small effects were found in this study for both breeds in each PRRS phase. However, none of the identified QTL overlapped between phases for either breed. This result was somewhat unexpected because genetic correlation estimates of reproductive traits between PRRS phases were generally high and positive, indicating similar genetic control for them, regardless of the PRRS phase. However, the power of detecting QTL in GWAS is impacted by the heritability of the trait and sample size. Thus, the low heritability estimates of these traits and the small sample size limited the identification of QTs for the same trait being identified between PRRS phases.

The number of identified QTL was much greater for the pre-PRRS phase than for the PRRS phase for both breeds. Although the number of animals used in the analyses were similar between these two phases, they were overall low. In addition, lowly heritable traits have a lower statistical power of GWAS to detect QTL, and thus, it could be that a larger dataset would result in more similar results between phases. Additionally, we were not able to identify specific SNPs that explained most of the %TGVM of the identified QTL. Most QTL identified in this study explained a low % TGVM of the traits, further supporting the general perception that reproductive traits are highly polygenic.

### Genomic Prediction Accuracies

Studies on genomics of response to PRRS have provided information on accuracies of genomic prediction but, to date, only results using growing piglets have been reported (Boddicker et al., 2014a; Waide et al., 2018). These authors reported high genomic prediction accuracies based on the WUR region associated with viremia and weight gain in pigs. To the best of our knowledge, our study is the first one to report GPAs of reproductive traits in PRRSV-infected sows.

Multiple scenarios were evaluated to perform genomic prediction of reproductive traits in PRRSV-infected sows. Due to the high r_g_ estimates for reproductive traits between pre-PRRS and PRRS phases, we evaluated how accuracies changed according to using data from only the PRRS phase, from only the pre-PRRS, or a combination of both. Furthermore, with the exception of GPS_pre–PRRS_, all analyses were performed using cross-validation (i) to avoid biased GPAs when using data from the PRRS phase for training SNP effects, and (ii) to better represent how genomic selection is done in practice. All these strategies resulted in a different number of animals used for training and validation, as seen in Supplementary Table 1. Finally, we used different statistical methods for genomic prediction; however, results were very similar across methods, further suggesting that no major QTL control the traits evaluated in this study. In general, there was not consistency in results according to GPSs across traits and breeds. Nonetheless, GPA results for Landrace were all positive and less variable compared to Duroc, which had large variation in GPAs with positive and negative values within traits and GPSs.

The GPAs of reproductive traits during a PRRS outbreak using marker estimates during the outbreak (i.e., GPS_PRRS_) were generally low to moderate. However, compared to the other GPSs, this scenario had overall lower variation in GPAs across folds. In Duroc, GPAs using GPS_PRRS_ were low and positive for TNB (GPA ± SD across folds = 0.32 ± 0.05), NBA (0.31 ± 0.01), and NBD (0.09 ± 0.06), and moderate and negative for NSB (−0.57 ± 0.04) and NBM (−0.46 ± 0.12). The only trait that had a substantial favorable GPA for this scenario in Duroc was NW, with 0.69 ± 0.06. In fact, this GPS was the best one for NW in Duroc. In Landrace, with the exception of TNB that had a very low GPA (0.09 ± 0.09), this scenario resulted in the largest or comparable GPAs for the other traits compared to the other GPSs. This scenario had the best GPA for NBA (0.37 ± 0.03), NBD (0.55 ± 0.12), and NBM (0.41 ± 0.14), and the second best for NSB (0.46 ± 0.08) and NW (0.44 ± 0.14). Therefore, genomic prediction of reproductive performance during a PRRS outbreak seems to be worthwhile in Landrace sows only.

In general, the r_g_ estimates of reproductive traits were moderate-high and positive between pre-PRRS and PRRS phases. Hence, we would expect high GPAs using GPS_pre–PRRS_ and GPS_pre–PRRS–4FCV_; however, this was not the case in these analyses. In Duroc, with the exception of NBA, all other traits had contrasting results between GPS_pre–PRRS_ and GPS_pre–PRRS–4FCV_, where GPAs were negative for one GPS and positive for the other. For example, training markers in the pre-PRRS phase using the same animals for training and validation (i.e., GPS_pre–PRRS_) was only beneficial for TNB (GPA = 0.60), whereas NBA had a very low GPA (∼0) and the other traits had substantially negative GPAs, ranging from −0.31 for NW to −1.03 for NBM. Interestingly, when a 4FCV was used when training markers in the pre-PRRS phase (i.e., GPS_Pre–PRRS–4FCV_), all results improved, with the exception for TNB (GPA = −0.39 ± 0.60). However, these remained negative for NBD (−0.03 ± 0.71) and NBM (−0.02 ± 1.93), and it was very low and positive for NW (0.02 ± 0.90). In contrast, the GPA for NSB in GPS_Pre–PRRS–4FCV_ was high and positive (0.84 ± 1.42), albeit very variable across folds. Finally, the GPA for NBA (0.31 ± 0.55) was the same level as in GPS_PRRS_ (0.31 ± 0.01), although the latter had a much lower variation across folds than the former. These results did not align with the r_g_ estimates of reproductive traits in Duroc between pre-PRRS and PRRS phases in Table 6. This could be due to the overall low sample size used in this study, which resulted in wide standard errors for the rg estimates, as well as large SD of GPAs across folds (Supplementary Table 2).

In Landrace, results between GPS_pre–PRRS_ and GPS_pre–PRRS–4FCV_ were much more consistent across traits. The only exception was for TNB. The GPA for this trait using GPS_pre–PRRS_ (1.16) was the highest across all traits and GPSs in Landrace. Although the GPA of TNB using GPS_pre–PRRS–4FCV_ was also high (0.77 ± 1.95), it was very variable across folds. For other traits, GPAs were moderate low for NBD, NSB, and NW, and close to zero for NBA and NBM. Moreover, these were consistently lower than the GPAs obtained when only the PRRS data were used for analyses (i.e., GPS_PRRS_). Contrary to what was seen in Duroc, the genomic prediction analyses of reproductive traits during a PRRS outbreak in Landrace sows were much more aligned with the r_g_ estimates between the pre-PRRS and PRRS phases in Table 6. Hence, phenotypic and genomic data from healthy sows could be used to promote improved reproductive performance during a PRRS outbreak.

The other two GPSs evaluated in this study (i.e., GPS_pre–PRRS,PRRS_ and GPS_pre–PRRS–4FCV,PRRS_) aimed to evaluate the use of data from both pre-PRRS and PRRS phases to predict reproductive performance during a PRRS outbreak. In both scenarios, marker estimates were obtained separately using data from the pre-PRRS and PRRS phases. Then, GEBVs in the validation sets were calculated as the average GEBV based on the estimates from each phase. This strategy was used because GWAS and r_g_ estimates results within a breed did not indicate that the genomic control of reproductive traits is the same between pre-PRRS and PRRS phases. Therefore, we expected that the results for GPS_pre–PRRS,PRRS_ would be a combination of the results based on GPS_PRRS_ and GPS_Pre–PRRS_, whereas for GPS_pre–PRRS–4FCV,PRRS_ would be a combination of the results based on GPS_PRRS_ and GPS_Pre–PRRS–4FCV_. In fact, this was observed in most cases.

In most analyses, GPAs using GPS_pre–PRRS–4FCV,PRRS_ were greater than using GPS_pre–PRRS,PRRS_. This is in accordance with the previous results shown for GPS_pre–PRRS–4FCV_, which had overall greater GPAs than for GPS_pre–PRRS_, especially in Duroc sows. In Duroc, these two GPSs had overall the best results across all GPSs. GPS_pre–PRRS–4FCV,PRRS_ resulted in the highest GPAs in Duroc for NBA (0.61 ± 0.48), NBD (0.55 ± 0.73), NSB (0.98 ± 2.05), and NBM (1.19 ± 2.55), whereas GPS_pre–PRRS,PRRS_ had the highest GPA for TNB (0.60 ± 0.12; which was the same as using GPS_pre–PRRS_). Among these results, the GPA for NBM was the only unexpected one since the GPAs for this trait using GPS_PRRS_ and GPS_Pre–PRRS_ were moderate to high and negative, with −0.46 ± 0.12 and −1.03, respectively. In addition, this analysis had the largest SD of GPAs across folds using BayesB, with 2.55. In fact, GPS_pre–PRRS–4FCV,PRRS_ had the overall greater variability in GPAs across folds (average SD of 1.24) in Duroc compared to all other GPS, followed by GPS_Pre–PRRS–4FCV_ (average SD = 1.02). Although this GPS resulted in overall better GPAs than all other GPS, this large variability in results suggests that such strategy might not be used to accurately obtain GEBVs for reproductive performance during a PRRS outbreak in Duroc.

In contrast, GPS_Pre–PRRS–4FCV_ had the second lowest variability in GPAs across folds (average SD = 0.49), behind only GPS_PRRS_ (average SD = 0.1). In general, differences in GPAs between GPS_pre–PRRS,PRRS_ and GPS_pre–PRRS–4FCV,PRRS_ were small. Furthermore, with the exception of TNB, results were consistently better than for GPS_Pre–PRRS_ and GPS_Pre–PRRS–4FCV_, and similar to those in GPS_PRRS_. Although GPAs for NSB (0.48 ± 0.29) and NW (0.45 ± 0.31) in GPS_pre–PRRS–4FCV,PRRS_ were numerically greater than in GPS_PRRS_ (0.46 ± 0.08 and 0.44 ± 0.14 for NSB and NW, respectively), the latter had a much lower GPA SD across folds than the former. This was also the case for the GPAs of the other traits that were similar between GPS_PRRS_ and these two GPS (GPS_pre–PRRS,PRRS_ and GPS_pre–PRRS–4FCV,PRRS_): (i) for NBA, GPAs were 0.37 ± 0.03 and 0.35 ± 0.27 for GPS_PRRS_ and GPS_pre–PRRS–4FCV,PRRS_, respectively; (ii) for NBD, GPAs were 0.55 ± 0.12 and 0.54 ± 0.31 for GPS_PRRS_ and GPS_pre–PRRS,PRRS_, respectively; and (iii) for NBM, GPAs were 0.41 ± 0.14 and 0.34 ± 0.51 for GPS_PRRS_ and GPS_pre–PRRS,PRRS_, respectively. Therefore, the marginal increase in GPAs when pre-PRRS data were used in combination with PRRS data for some of the traits does not seem to offset the greater variability in GPAs using this strategy.

The genomic prediction results presented in this study indicate that reproductive performance under a PRRS outbreak can be improved through genomic selection. However, Duroc results were highly variable across GPSs and traits, without a clear pattern, indicating that additional research is needed to evaluate the use of genomic selection for improved reproductive performance under a PRRS outbreak for this breed. However, it is important to note that this is a terminal breed, and hence, little emphasis is put on maternal traits in its selection index. In contrast, results for Landrace were more consistent. In general, using only data from the PRRS phase had similar results to those in which GEBVs were based on those obtained from the separate analyses using the pre-PRRS and PRRS phases. However, the high variability in GPAs when the data were combined does not support the use of this strategy to promote genetic gains for reproductive performance under a PRRS outbreak. Hence, the use of PRRS data only to train marker estimates is indicated. Nonetheless, additional strategies should be exanimated in the future, such as combining both pre-PRRS and PRRS phases when estimating marker effects. However, this strategy assumes that marker estimates are the same in both phases. Although this is a strong assumption, the overall high r_g_ estimates of reproductive performance between the pre-PRRS and PRRS phases indicate that there is potential in using this strategy to increase the size of the training set, which should then increase the accuracy of the marker estimates. Nonetheless, the proportion of data from each phase used in the training set should impact these results.

## Conclusion

Our results indicate that heritabilities are overall low for most reproductive traits, regardless of PRRS-phase. The high genetic correlations with reproductive traits between PRRS phases suggest that selection for improved reproductive performance in a clean environment (i.e., in the absence of PRRS) would improve response during a PRRS outbreak, but with limited efficiency due to their low heritability estimates. Thus, an indicator trait that we can indirectly use to increase the response to selection for these traits is then desirable. Our results also indicate that, phenotypically, Duroc sows are less impacted by PRRS than Landrace sows, indicating that they have overall greater PRRS resilience than Landrace sows. The MARC0034894 SNP previously associated with NSB during a PRRSV infection was associated with most traits in our study. Associations between this SNP and reproductive performance were found depending on the trait, breed, and PRRS phase. Nonetheless, results indicate that this marker has the potential to be used to improve reproductive performance. In contrast, the lack of substantial associations between the WUR10000125 SNP with reproductive performance does not support the use of this marker for reproductive performance. Genomic analyses showed that several QTL control reproductive performance, most of them explaining a very small percentage of the additive genetic variance, indicating that these traits are highly polygenic. Our study is the first one to provide genomic prediction accuracies for reproductive traits during a PRRS outbreak. Although results were overall variable in Duroc, those from Landrace indicate that genomic selection for improved reproductive performance during a PRRS outbreak might be more accurate by training markers using data from PRRSV-infected sows. Overall, this study helped to understand better the genetic basis of PRRS response to potentially improve the reproductive performance of sows.

## Data Availability Statement

The datasets presented in this article are not readily available because the data that support the findings of this study are not publicly available. Data may be available from authors upon reasonable request and authorization from the company that generated the data.

## Ethics Statement

The animal study was reviewed and approved by the Institutional Animal Care and Use Committee (IACUC) at Iowa State University (protocol number 6-17-8551-S). Written informed consent was obtained from the owners for the participation of their animals in this study.

## Author Contributions

FH performed statistical analyses, interpreted results, prepared figures and tables, and drafted the manuscript. JB, LK, and JD were involved in the interpretation and discussion of results. YH and KG provided the data, led the collection of blood samples, and interpreted results. LS provided help and guidance for statistical analyses, being involved in the discussion of results as well. NS assisted with data analysis, interpretation of results, and drafted the manuscript. All authors read and approved the final version of the manuscript.

## Conflict of Interest

The authors declare that this study received funding from Smithfield Premium Genetics, NC, United States. The funder had the following involvement with the study: providing performance and genotype data and collection of blood samples. In addition, YH and KG are employed by the company Smithfield Premium Genetics, NC, United States. The remaining authors declare that the research was conducted in the absence of any commercial or financial relationships that could be construed as a potential conflict of interest.

## Publisher’s Note

All claims expressed in this article are solely those of the authors and do not necessarily represent those of their affiliated organizations, or those of the publisher, the editors and the reviewers. Any product that may be evaluated in this article, or claim that may be made by its manufacturer, is not guaranteed or endorsed by the publisher.

## Abbreviations

*APOBEC3B*: apolipoprotein B mRNA editing enzyme catalytic subunit 3B gene
*EIF3L*: eukaryotic translation initiation factor 3 subunit L gene
FYW: Farrow-year-week
GEBV: genomic estimated breeding value
*GLIPR1L1*: GLIPR1-like protein 1 gene
*GLIPR1L2*: GLIPR1-like protein 2 gene
GPA: genomic prediction accuracy
GPS: genomic prediction scenarios
GWAS: genome-wide association studies
MARC: MARC0034894
Mb: megabases
MCMC: Markov Chain Monte Carlo
NBA: number of piglets born alive
NBA_TNB_: number of piglets born alive with total number of piglets born used as the offset
NBD: number of piglets born dead
NBD_TNB_: number of piglets born dead with total number of piglets born used as the offset
NBM: number of mummified piglets
NBM_TNB_: number of mummified piglets with total number of piglets born used as the offset
NSB: number of stillborn piglets
NSB_TNB_: number of stillborn piglets with total number of piglets born used as the offset
NW: number of piglets weaned
NW_TNB,XF_: number of piglets weaned with total number of piglets born and number of cross-fostered pigs used as the offset
NW_NBA,XF_: number of piglets weaned with number of piglets born alive and number of cross-fostered pigs used as the offset
*PDGFB*: platelet-derived growth factor subunit B gene
PRRS: porcine reproductive and respiratory syndrome
PRRSV: porcine reproductive and respiratory syndrome virus
QTL: quantitative trait loci
SNP: single nucleotide polymorphisms
*SOX10*: SRY-box transcription factor 10 gene
*SPATA17*: spermatogenesis associated 17 gene
SSC: *Sus scrofa* chromosome
TGVM: total additive genetic variance accounted for by markers
TNB: total number of piglets born
XF: net number of cross-fostered pigs
XV: cross-validation
WUR: WUR10000125 SNP.
