# Supplementary material for: Host Genetics of Response to Porcine Reproductive and Respiratory Syndrome in Sows: Reproductive Performance
Source: Front Genet. 2021 Aug 4;12:707870. doi: 10.3389/fgene.2021.707870 (PMC8371709; doi:10.3389/fgene.2021.707870)
Supplement: Supplementary file 1 [file Data_Sheet_1.pdf]

Host Genetics of Response to Porcine Reproductive and Respiratory Syndrome in Sows: Reproductive Performance by Hickmann et al. (2021)

Supplementary Materials

Supplementary table 1. Number of records used for training and validation in each fold (F) across genomic prediction scenarios (GPS)[1] of reproductive traits[2] during a Porcine Reproductive and Respiratory Syndrome (PRRS) outbreak by breed.

GPS$_{PRRS}$

	**Duroc**								**Landrace**							
	Training (PRRS)				*Validation*				*Training (PRRS)*				*Validation*			
Trait	F1	F2	F3	F4	F1	F2	F3	F4	F1	F2	F3	F4	F1	F2	F3	F4
TNB	372	372	372	372	124	124	124	124	321	321	321	321	107	107	107	107
NBA	372	372	372	372	124	124	124	124	321	321	321	321	107	107	107	107
NBD	315	315	315	315	105	105	105	105	275	275	275	276	92	92	92	91
NSB	315	315	315	315	105	105	105	105	275	275	275	276	92	92	92	91
NBM	309	312	308	310	104	101	105	103	275	275	275	276	92	92	92	91
NW	372	372	372	372	124	124	124	124	321	321	321	321	107	107	107	107

GPS$_{pre\text{-}PRRS}$

	**Duroc**		**Landrace**	
Trait	*Training (Pre-PRRS)*	*Validation*	*Training (Pre-PRRS)*	*Validation*
TNB	478 (239)[3]	496 (257)[4]	459 (213)[3]	428 (215)[4]
NBA	478 (239)[3]	496 (257)[4]	461 (213)[3]	428 (215)[4]
NBD	475 (239)[3]	420 (181)[4]	459 (213)[3]	367 (154)[4]
NSB	478 (239)[3]	420 (181)[4]	461 (213)[3]	367 (154)[4]
NBM	475 (239)[3]	413 (174)[4]	459 (213)[3]	367 (154)[4]
NW	478 (239)[3]	496 (257)[4]	461 (213)[3]	428 (215)[4]

GPS$_{pre\text{-}PRRS\text{-}4FCV}$

	**Duroc**								**Landrace**							
	Training (Pre-PRRS)				*Validation*				*Training (Pre-PRRS)*				Validation			
Trait	F1	F2	F3	F4	F1	F2	F3	F4	F1	F2	F3	F4	F1	F2	F3	F4
TNB	432 (239)[5]	432 (239)[5]	432 (239)[5]	431 (239)[5]	124	124	124	124	375 (213)[5]	374 (213)[5]	374 (213)[5]	374 (213)[5]	107	107	107	107
NBA	432 (239)[5]	432 (239)[5]	432 (239)[5]	431 (239)[5]	124	124	124	124	375 (213)[5]	374 (213)[5]	374 (213)[5]	374 (213)[5]	107	107	107	107
NBD	375 (239)[5]	375 (239)[5]	375 (239)[5]	374 (239)[5]	105	105	105	105	329 (213)[5]	328 (213)[5]	328 (213)[5]	329 (213)[5]	92	92	92	91
NSB	375 (239)[5]	375 (239)[5]	375 (239)[5]	364 (239)[5]	105	105	105	105	329 (213)[5]	328 (213)[5]	328 (213)[5]	329 (213)[5]	92	92	92	91
NBM	370 (239)[5]	370 (239)[5]	369 (239)[5]	369 (239)[5]	104	101	105	103	329 (213)[5]	328 (213)[5]	328 (213)[5]	329 (213)[5]	92	92	92	91
NW	432 (239)[5]	432 (239)[5]	432 (239)[5]	431 (239)[5]	124	124	124	124	375 (213)[5]	374 (213)[5]	374 (213)[5]	374 (213)[5]	107	107	107	107

$GPS_{pre\text{-}PRRS,PRRS}$

	Duroc									Landrace								
	Training					*Validation*				*Training*					*Validation*			
		PRRS								*Pre-PRRS*	*PRRS*							
Trait	*Pre-PRRS*	F1	F2	F3	F4	F1	F2	F3	F4		F1	F2	F3	F4	F1	F2	F3	F4
TNB	478 (239)[3]	372	372	372	372	124	124	124	124	459 (213)[3]	321	321	321	321	107	107	107	107
NBA	478 (239)[3]	372	372	372	372	124	124	124	124	461 (213)[3]	321	321	321	321	107	107	107	107
NBD	475 (239)[3]	315	315	315	315	105	105	105	105	459 (213)[3]	275	275	275	276	92	92	92	91
NSB	478 (239)[3]	315	315	315	315	105	105	105	105	461 (213)[3]	275	275	275	276	92	92	92	91
NBM	475 (239)[3]	309	312	308	310	104	101	105	103	459 (213)[3]	275	275	275	276	92	92	92	91
NW	478 (239)[3]	372	372	372	372	124	124	124	124	461 (213)[3]	321	321	321	321	107	107	107	107

$GPS_{pre\text{-}PRRS\text{-}4FCV,PRRS}$

	Duroc												Landrace											
	Training								*Validation*				*Training*								*Validation*			
	Pre-PRRS				*PRRS*								*Pre-PRRS*				*PRRS*							
Trait	F1	F2	F3	F4	F1	F2	F3	F4	F1	F2	F3	F4	F1	F2	F3	F4	F1	F2	F3	F4	F1	F2	F3	F4
TNB	432 (239)[5]	432 (239)[5]	432 (239)[5]	431 (239)[5]	372	372	372	372	124	124	124	124	375 (213)[5]	374 (213)[5]	374 (213)[5]	374 (213)[5]	321	321	321	321	107	107	107	107
NBA	432 (239)[5]	432 (239)[5]	432 (239)[5]	431 (239)[5]	372	372	372	372	124	124	124	124	375 (213)[5]	374 (213)[5]	374 (213)[5]	374 (213)[5]	321	321	321	321	107	107	107	107
NBD	375 (239)[5]	375 (239)[5]	375 (239)[5]	374 (239)[5]	315	315	315	315	105	105	105	105	329 (213)[5]	328 (213)[5]	328 (213)[5]	329 (213)[5]	275	275	275	276	92	92	92	91
NSB	375 (239)[5]	375 (239)[5]	375 (239)[5]	364 (239)[5]	315	315	315	315	105	105	105	105	329 (213)[5]	328 (213)[5]	328 (213)[5]	329 (213)[5]	275	275	275	276	92	92	92	91
NBM	370 (239)[5]	370 (239)[5]	369 (239)[5]	369 (239)[5]	309	312	308	310	104	101	105	103	329 (213)[5]	328 (213)[5]	328 (213)[5]	329 (213)[5]	275	275	275	276	92	92	92	91
NW	432 (239)[5]	432 (239)[5]	432 (239)[5]	431 (239)[5]	372	372	372	372	124	124	124	124	375 (213)[5]	374 (213)[5]	374 (213)[5]	374 (213)[5]	321	321	321	321	107	107	107	107

[1]GPS described in the Materials and Methods section and in Table 2 of the manuscript;

[2]TNB, total number of piglets born; NBA, number of piglets born alive; NBD, number of piglets born dead; NSB, number of stillborn piglets; NBM, number of mummified piglets; NW, number of piglets weaned;

[3]Number of animals with pre-PRRS data included in the training dataset that did not have PRRS data included in the validation dataset;

[4]Number of animals with PRRS data included in the validation dataset that did not have pre-PRRS data included in the training dataset;

[5]Number of animals with pre-PRRS data included in the training dataset for this fold that did not have PRRS data included in the validation dataset.

Supplementary table 2. Genomic prediction accuracies[1] of reproductive traits[2] during a Porcine Reproductive and Respiratory Syndrome (PRRS) outbreak by breed and Bayesian method across genomic prediction scenarios (GPS)[3].

GPS $\boldsymbol{GPS_{PRRS}}$

	**Duroc**			**Landrace**		
Trait[1]	*BayesB*	*BayesC*	*BayesC0*	*BayesB*	*BayesC*	*BayesC0*
TNB	0.32 (0.05)	0.27 (0.05)	0.27 (0.04)	0.09 (0.09)	0.09 (0.09)	0.07 (0.06)
NBA	0.31 (0.01)	0.33 (0.02)	0.34 (0.02)	0.37 (0.03)	0.38 (0.04)	0.37 (0.04)
NBD	0.09 (0.06)	0.09 (0.05)	0.08 (0.06)	0.55 (0.12)	0.56 (0.12)	0.55 (0.12)
NSB	-0.57 (0.04)	-0.61 (0.04)	-0.66 (0.04)	0.46 (0.08)	0.47 (0.08)	0.47 (0.08)
NBM	-0.46 (0.12)	-0.45 (0.12)	-0.45 (0.12)	0.41 (0.14)	0.40 (0.14)	0.42 (0.14)
NW	0.69 (0.06)	0.65 (0.06)	0.64 (0.05)	0.44 (0.14)	0.43 (0.14)	0.43 (0.14)

$\boldsymbol{GPS_{pre\text{-}PRRS}}$

	**Duroc**			**Landrace**		
Trait	*BayesB*	*BayesC*	*BayesC0*	*BayesB*	*BayesC*	*BayesC0*
TNB	0.60	0.30	0.31	1.16	1.09	1.08
NBA	~0	~0	-0.01	0.01	0.07	0.07
NBD	-0.63	-0.57	-0.52	0.37	0.38	0.40
NSB	-0.50	0.14	0.15	0.40	0.45	0.48
NBM	-1.03	-0.60	-0.60	0.07	0.03	0.03
NW	-0.31	-0.17	-0.17	0.29	0.32	0.32

$\boldsymbol{GPS_{pre\text{-}PRRS\text{-}4FCV}}$

	**Duroc**			**Landrace**		
Trait	*BayesB*	*BayesC*	*BayesC0*	*BayesB*	*BayesC*	*BayesC0*
TNB	-0.39 (0.60)	-0.36 (0.58)	-0.36 (0.58)	0.77 (1.95)	0.75 (1.91)	0.77 (1.97)
NBA	0.31 (0.55)	0.29 (0.54)	0.25 (0.51)	0.03 (0.57)	0.04 (0.52)	0.05 (0.50)
NBD	-0.03 (0.71)	-0.02 (0.73)	-0.07 (0.63)	0.37 (0.17)	0.39 (0.16)	0.41 (0.16)
NSB	0.84 (1.42)	0.92 (1.39)	0.93 (1.42)	0.37 (0.23)	0.43 (0.23)	0.45 (0.23)
NBM	-0.02 (1.93)	-0.02 (1.88)	0.02 (1.95)	0.02 (0.32)	-0.01 (0.28)	-0.01 (0.28)
NW	0.02 (0.90)	0.03 (0.91)	0.04 (0.93)	0.32 (0.09)	0.28 (0.11)	0.28 (0.09)

$\boldsymbol{GPS_{pre\text{-}PRRS,PRRS}}$

	Duroc			Landrace		
Trait	*BayesB*	*BayesC*	*BayesC0*	*BayesB*	*BayesC*	*BayesC0*
TNB	0.60 (0.12)	0.28 (0.14)	0.27 (0.14)	0.34 (2.08)	0.27 (2.20)	0.23 (2.29)
NBA	0.40 (0.46)	0.29 (0.20)	0.30 (0.19)	0.23 (0.62)	0.32 (0.54)	0.32 (0.52)
NBD	0.28 (0.60)	0.26 (0.59)	0.27 (0.60)	0.54 (0.31)	0.54 (0.30)	0.54 (0.29)
NSB	-0.27 (0.25)	-0.31 (0.44)	-0.55 (0.36)	0.44 (0.25)	0.46 (0.22)	0.48 (0.23)
NBM	-0.12 (0.51)	-0.18 (0.76)	-0.15 (0.78)	0.34 (0.51)	0.37 (0.48)	0.38 (0.48)
NW	0.38 (0.19)	0.27 (0.18)	0.27 (0.18)	0.41 (0.26)	0.44 (0.39)	0.44 (0.38)

GPS$_{pre\text{-}PRRS\text{-}4FCV,PRRS}$

	Duroc			Landrace		
Trait	*BayesB*	*BayesC*	*BayesC0*	*BayesB*	*BayesC*	*BayesC0*
TNB	0.32 (0.68)	0.46 (0.76)	0.50 (0.67)	0.41 (1.19)	0.32 (1.24)	0.30 (1.31)
NBA	0.61 (0.48)	0.65 (0.46)	0.67 (0.45)	0.35 (0.27)	0.38 (0.25)	0.38 (0.22)
NBD	0.55 (0.73)	0.44 (0.68)	0.63 (0.92)	0.49 (0.37)	0.50 (0.37)	0.49 (0.38)
NSB	0.98 (2.05)	1.15 (2.10)	0.78 (1.85)	0.48 (0.29)	0.53 (0.31)	0.55 (0.32)
NBM	1.19 (2.55)	1.17 (2.85)	1.27 (2.81)	0.24 (0.51)	0.30 (0.47)	0.32 (0.47)
NW	0.44 (0.93)	0.33 (1.08)	0.29 (1.07)	0.45 (0.31)	0.45 (0.43)	0.45 (0.42)

[1]Numbers in parenthesis represent the standard deviation of genomic prediction accuracies across the 4 folds;
[2]TNB, total number of piglets born; NBA, number of piglets born alive; NBD, number of piglets born dead; NSB, number of stillborn piglets; NBM, number of mummified piglets; NW, number of piglets weaned;
[3]GPS described in the Materials and Methods section and in Table 2 of the manuscript.
